# Mass Spectral Profile for Rapid Differentiating Beta-Lactams from Their Ring-Opened Impurities

**DOI:** 10.1155/2015/697958

**Published:** 2015-05-18

**Authors:** Hecheng Wang, Haiwei Huang, Jin Cao, Dehua Chui, Shengyuan Xiao

**Affiliations:** ^1^School of Life Science, Beijing Institute of Technology, 5 Zhongguancun Street, South Haidian District, Beijing 100081, China; ^2^Neuroscience Research Institute, Health Science Center, Peking University, Beijing 100191, China; ^3^National Institutes for Food and Drug Control, Beijing 100050, China

## Abstract

High performance liquid chromatography tandem mass spectrometry (HPLC MS) has been widely used for *β*-lactam antibiotics determination. However, its application to identify impurities of these frequently used drugs is not sufficient at present. In this job, characteristic profiles of the collision induced dissociation (CID) spectra of both *β*-lactams and ring-opened *β*-lactams were extracted from the MS data of six *β*-lactam antibiotics and their forty-five impurities, and were confirmed by the MS data reported in the literature. These characteristics have been successfully applied to rapid differentiation of *β*-lactam and ring-opened *β*-lactam impurities in cefixime, cefdinir, and cefaclor. However, these characteristic profiles can only be obtained under low activating voltage. They did not display in the high energy activated CID spectra. Diagnostic fragmentations for determining the localization of double bond and substituents on the thiazine ring and the side chain were also observed. In addition, several characteristic fragmentations are hopeful to be used to differentiate the configurations of C-2 on the thiazine ring of ring-opened impurities, which is generally disadvantageous of mass spectrometry. Taken together, forty-five impurities were identified from the capsules of cefixime, cefdinir, and cefaclor.

## 1. Introduction

Health concerns have increased the regulatory stringency on the control of impurities and degradation products not only in new drug substances and its finished pharmaceutical formulations but also in the existing drug in recent years [1]. *β*-Lactam antibiotics are among the most frequently used drugs for infectious disease treatment. Identification and characterization of impurities and degradants of *β*-lactam antibiotics are important for public health. Related substances test of *β*-lactam antibiotics has been required by official compendia like EP and USP. The core structure of a *β*-lactam antibiotic is a fused *β*-lactam thiazole or thiazine ring system. Because the strained *β*-lactam ring is susceptible to cleavage [2, 3], the degradation of the drug was suggested to be a major hurdle in the development and application of this kind of drugs [4]. Nowadays, antibiotic-resistance caused by antibiotics abuse is becoming a public health problem [5]. Identification and characterization of impurities and of *β*-lactam antibiotics are important not only in drug substance but also in the meat [6–14], the milk [15], and the environment [7]. In accordance with the requirements of ICH (International Conference on Harmonization) [16], the impurity profile study has to be carried out to identify and characterize all the unknown impurities. To profile the impurities in a *β*-lactam antibiotic, the impurities are generally isolated from treated drug substance or synthesized then identified with NMR and spectrometry method [17]. This method can obtain unequivocal structure of the impurities. However, it is tedious and time consuming, especially for the low abundant impurities. Rapid and sensitive method is demanded for the aim of *β*-lactams impurities profiling.

As a sensitive and rapid method for determination and identification of chemical compound from complex samples, high performance liquid chromatography tandem mass spectrometry (HPLC MS) has been used as a powerful method for *β*-lactam antibiotics determination [15]. So far as we know, there were five impurities of cefixime in drug substance [16, 18] and six degradation products of cephapirin in bovine milk [19] which are identified based on the data of HPLC MS. Holstege et al. discussed the CID characteristics of six cephalosporins in negative mode [18, 19]. However, only few applications of this method to identified impurities of *β*-lactam antibiotics were reported. Hence it is worthwhile to extract diagnostic characteristics from MS data of compounds with structural diversity and develop methods for impurities identification based on HPLC MS data of *β*-lactam antibiotics and related samples. The relationships between fragmentation channels and structural characters, especially for the impurities, have seldom been discussed. Herein, we described a rapid method to differentiate ring-opened *β*-lactam antibiotics impurities from those with the unbroken lactam ring. To develop this method, the collision induced dissociation (CID) spectra of several kinds of *β*-lactam antibiotics and impurities were compared to extract their diagnostic fragmentations. To the best of our knowledge, this is the first report on the overall CID character for differentiation of the ring-opened and unopened impurities of *β*-lactam antibiotics. Some diagnostic fragmentations for the localization of the double bond on the thiazine ring, substituent groups on the side chain, as well as the configuration of C-2 of the thiazine ring on ring-opened impurities, were also discussed.

## 2. Materials and Methods

### 2.1. Reagents and Chemicals

Capsules of amoxicillin and five cephalosporins (cefixime, cefdinir, cefprozil, cefaclor, and cefuroxime) were bought from local pharmacy. HPLC grade methanol and acetonitrile were purchased from Fisher Company, USA. Formic acid (HPLC grade) was produced by Beijing Fine Chemical Co., Ltd.

### 2.2. Apparatus

A HPLC Q-TOF system equipped with a binary pump (G1312A), a degasser (G1379B), an autosampler (G1367C), and a Q-TOF MS (6520) with an ESI ion source (Agilent, USA) was employed for high energetic collision induced dissociation analysis. The data acquisition and processing were performed by Mass Hunter software (Agilent, USA).

Low energetic collision induced dissociation analysis was satisfied by busing a HPLC trap system consisting of a quaternary pump (G1311A), a degasser (G1379A), an autosampler (G1313A), a diode array detector (G1315B), and a trap mass spectrometer (SL) with an ESI ion source. The HPLC system was controlled by ChemStation B 10.01. The trap mass spectrometer was controlled by trap 5.3. The ultraviolet data was analyzed by ChemStation B 10.01. The mass spectrometry data processing was performed by Data Analysis 5.3.

### 2.3. Methods

#### 2.3.1. Sample Preparation

The content of *β*-lactam in the capsule was taken out and suspended in a solution (containing 70% water, 29% methanol, and 1% acetic acid, v/v) by votex for 10 min. The mixture was centrifuged for 10 min at 1000 rpm. 10 *μ*L of the supernatant was injected to HPLC MS for analysis.

#### 2.3.2. Separation

The separation of all *β*-lactams, except for cefprozil, was achieved using an Agilent Eclipse XDB-C18 column (2.1 × 150 mm, 5 *μ*m). The mobile phase consists of water (A) and acetonitrile (B), both containing 0.1% acetic acid (v/v). The gradient was as follows: 10% B was maintained at the first 3 min, then a linear gradient of 10–20% B for 3–10 min, and 20–50% B for 10–30 min; this composition was maintained from 30 to 35 min and then returned to the initial condition in 5 minutes. The flow rate of the mobile phase was 0.3 mL/min. The chromatograms were recorded at 230, 254, and 265 nm for each run. The ultraviolet spectra ranged from 190 to 400 nm, with 2 nm step.

The separation of cefprozil was achieved on a pentafluorophenyl bonded silica gel column (4.6 × 250 mm, 5 *μ*m, Discovery Sciences). The mobile phases are the same as abovementioned. The gradient was as follows: 10% was also maintained at the first 3 minutes, then a linear gradient of 10–20% B for 3–10 min, 20–35% B for 10–35 min, and 35–60% B for 35–50 min; this composition was maintained from 50 to 55 min and then returned to the initial condition in 5 minutes. The flow rate of the mobile phase was 0.6 mL/min.

#### 2.3.3. Mass Spectrometry Analysis

Approximately 200 *μ*L eluent was loaded to the mass spectrometer per min for all ESI MS/MS analyses. For low energy activated CID experiments, the interface parameter was set as follows: the pressure of nebulizer gas was 28 psi; the flow of dry gas was 8 L/min; and the temperature dry gas was 325°C. The ion trap parameter was set as a smart mode: target, 400 amu (atom mass unit); compound stability, 100%; and trap drive level, 80%. The scan range was 50 to 800 amu. The maximum ion count accumulated in the trap was set as 30000. The maximum accumulation time was set as 50 milliseconds. The MS/MS experiment was performed in an auto MS/MS mode. One precursor was selected at each scan cycle; each precursor was excluded after 3 spectra. The excitation amplitude voltage was 1 electro valve. The excitation range was 10 amu. The excitation time was 40 milliseconds. The cut-off scan range for MS/MS was set as 27%.

For high energy excitated CID analyses, the pressure of nebulizer gas was 20 psi. The flow of dry gas was 10 L/min. Its temperature was set as 350°C. The fragmentation amplitude voltage was 100 volt. The skimmer voltage difference was 65 volt. The MS/MS experiment was performed in an auto MS/MS mode. The excitation amplitude was 26 volt. The* m/z* value range of precursors was 200 to 800. The daughter ion* m/z* values ranged from 50 to 1200.

## 3. Results and Discussion

### 3.1. Fragmentation Patterns of Cefixime Activated under Low Resonance Voltage

**Figure 1:**
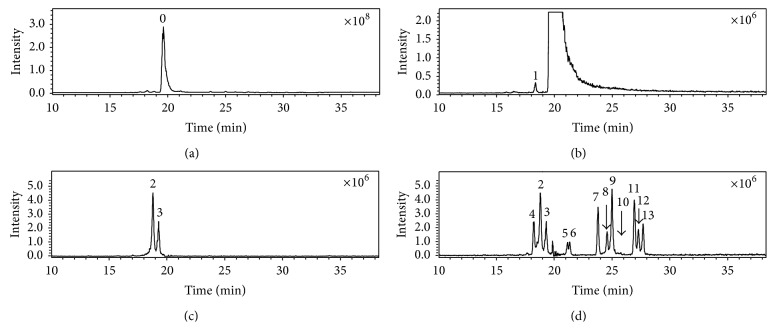
Representative chromatograms of cefixime and impurities. (a) Total ion chromatogram of cefixime capsule (TIC), (b) extracted ion chromatogram (EIC) at* m/z* 454; (c) EIC at* m/z* 472; (d) EIC exclude* m/z* 454. 0, cefixime; 1,* m/z* 454; 2, 3,* m/z* 472; 4,* m/z* 442; 5, 6,* m/z* 428; 7,* m/z* 468; 8, 9,* m/z* 486; 10,* m/z* 442; 11,* m/z* 482; and 12, 13,* m/z* 500. The data was recorded under positive mode with an electrospray ionization source (ESI). The scan range is between 100 amu and 800 amu.

**Figure 2:**
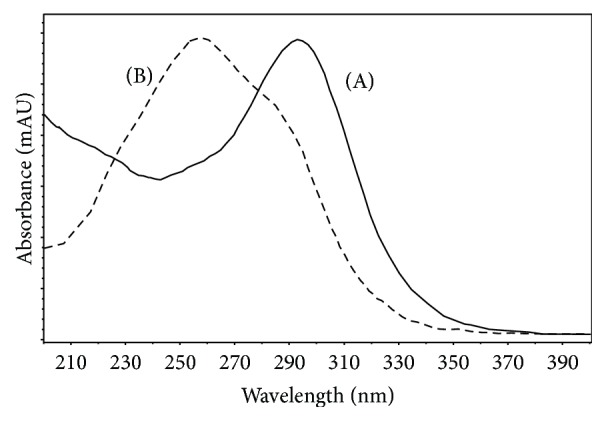
Represent online ultra violine spectrum (UV) of cefixime and ring-opened cefixime. The spectrum was recorded online. The solution is acetonitrile (32–36%).

Figure 1 shows the representative chromatogram of cefixime capsule and the extracted ion chromatograms (EIC) of its impurities.  Figure 2(A) represents the online recorded UV spectrum of cefixime (compound 0), and the absorption maximum at 295 nm corresponds to the structure of 7-aminothiazin lactam [20, 21]. The absorption of the thiazole ring in the molecule (approximately 260 nm) was overlapped by this peak.

**Scheme 1:**
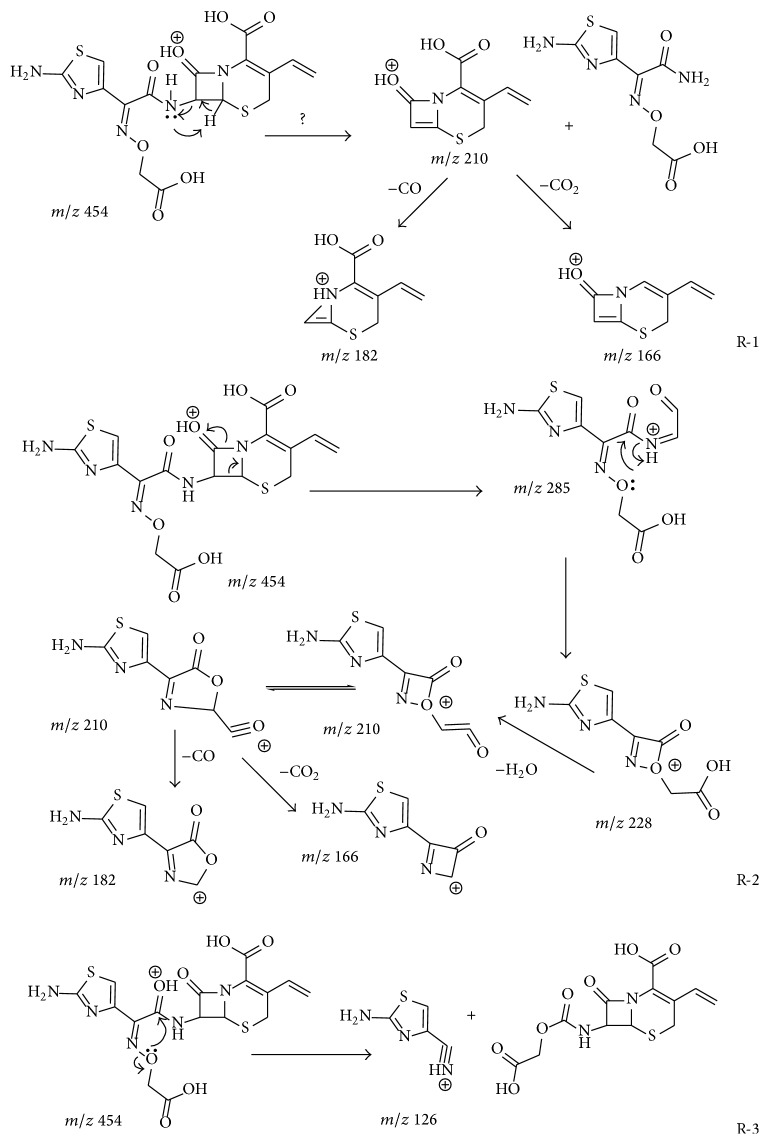
Proposed fragmentation pattern for cefixime.

**Figure 3:**
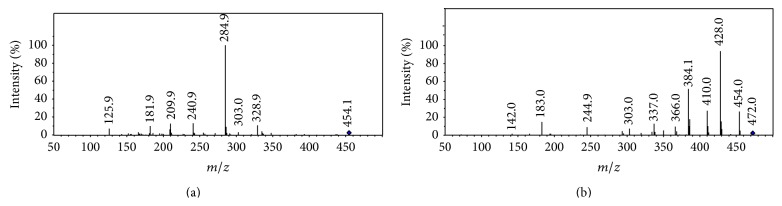
Representative low energy activated CID spectra of cefixime (a) and ring-opened cefixime (b).

**Scheme 2:**
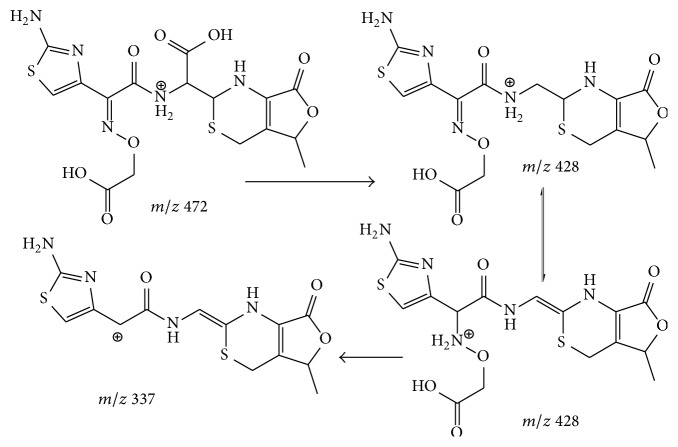
Proposed mechanism for the formation of daughter ion* m/z* 337.

**Scheme 3:**
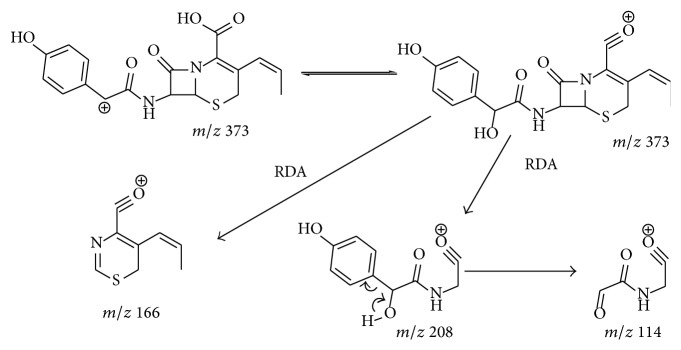
Fragmentation patterns proposed for deaminated daughter ion of cefprozil (R-1) and amoxillin (R-2).

The positive ESI MS/MS spectrum of protonated cefixime (*m/z* 454) is shown in Figure 3(a). Its CID spectrum displayed a prominent fragment (*m/z* 285) that originates from the cleavage of the *β*-lactam ring. Other fragmentations, for example, loss of CO_2_, were observed in very low yields (less than 20%). Dehydrated fragment and decarboxylated fragment were displayed in the CID spectrum at* m/z* 436 and* m/z* 410, respectively. The fragment cation* m/z* 241 was assumed to correspond to the elimination of carbon dioxide from the cation at* m/z* 285. The peak at* m/z* 210 was primarily suspected to be structurally related to thiazine lactam ring, originating from the cleavage of C(7)-N bond localized on the *β*-lactam ring (Scheme 1, R-1). The expected extensive fragmentations of losing a carbon monoxide (*m/z* 182) or a carbon dioxide (*m/z* 166) was also observed. However, this fragment, as well as the fragment ion* m/z* 182, was also observed in the CID spectra of all *β*-lactam impurities related to cefixime, including compound 4 whose substituent group on the thiazine ring is different from cefixime (see Figure S-1 Supplementary Material available online at http://dx.doi.org/10.1155/2015/697958). Hence, the fragment originating from the other part of the molecule (side chain) cannot be ruled out. This fragment ion has been assumed to be formed from the cleavage of the thiazole ring of the cation at* m/z* 285 in the literature [19]. However, the cation formed from this fragmentation channel contains two nitrogen atoms. Its* m/z* value was 221 but not 210 according to the nitrogen rule of an even-electron ion. The oxygen atom of an oxime is generally electron rich and is ready to transfer to an electron deficient carbon. Therefore, a transesterification reaction is proposed to explain the mechanism of the fragment* m/z* 210 formation in Scheme 1, R-2. The fragment ion* m/z* 126 is believed to originate from the transesterification rearrangement of the side chain of cefixime (Scheme 1, R-3), because it was also observed in the CID spectrum of cefdinir (Figure S-2), as well as most impurities related cefixime (Figures S-1 and S-3). The fragment ion* m/z* 329 might be responsible for the RDA (retro-Diels Alder) cleavage of the thiazole ring (Scheme S-1).

The difference among the impurities related to cefixime is usually the substituent groups localized on the thiazine ring or the oxime. The main fragment formed from the cleavage of *β*-lactam ring may sever as a diagnostic character to determine the localization of these substituent groups thereof. The observation of other fragments for example,* m/z* 210,* m/z* 182, and so forth, is helpful to confirm of the determination.

### 3.2. Fragmentation Patterns of Ring-Opened Cefixime Activated under Low Resonance Voltage

The identification of ring-opened cefixime was based on the UV spectrum (Figure 2) and mass data (Figure 3). There are two compounds observed in the EIC of cefixime capsule at* m/z* 472 (Figure 1(c), compounds 2 and 3).  Figure 2(B) represents the online UV spectrum of compounds 2 and 3. The absorption maximum at 280 nm corresponds to the structure of thiazine ring. Blue shift and hyperchromic effect observed are responsible for the reduced degree of conjugation. Pseudomolecular ion at* m/z *472 indicated that the molecular weights (Mw) of compounds 2 and 3 were both 471. The observation of prominent fragments at* m/z* 428 [M+H–CO_2_]^+^,* m/z* 384 [M+H–CO_2_–CO_2_]^+^,* m/z* 410 [454–CO_2_]^+^, and* m/z* 366 [454–CO_2_–CO_2_]^+^ in their CID spectra confirmed that the *β*-lactam ring, which is more prone to dissociation, was opened. Based on these observations, compounds 2 and 3 were identified as the stereoisomers of cefixime impurity A, 2-((Z)-2-(2-aminothiazol-4-yl)-2-(carboxymethoxyimino)acetamido)-2-((2R,5R/S)-5-methyl-7-oxo-2,4,5,7-tetrahydro-1H-furo[3,4-d][1,3]thiazin-2-yl) acetic acid. For the absence of unstable *β*-lactam ring in the structure, fragments displayed in the CID spectrum of a ring-opened impurity related to *β*-lactam are mainly those originated from elimination of small molecules, for example, H_2_O, CO_2_, and so forth; similar CID spectra have also been displayed by many ring-opened *β*-lactams. Besides the prominent fragments, daughter ion* m/z* 337 was observed in almost all ring-opened impurities of cefixime. Similar to the elimination of NH_3_ from cefprozil and amoxillin, the formation of fragment at* m/z* 337 was proposed through the mechanism in Scheme 3. The stabilization of the product cation by the expansion of conjugation system was assumed to be the driving force of the fragmentation. The formation of fragment at* m/z* 337 was also observed in CID of cefdinir.

### 3.3. Characteristic Profiles of *β*-Lactams and Ring-Opened *β*-Lactams

**Figure 4:**
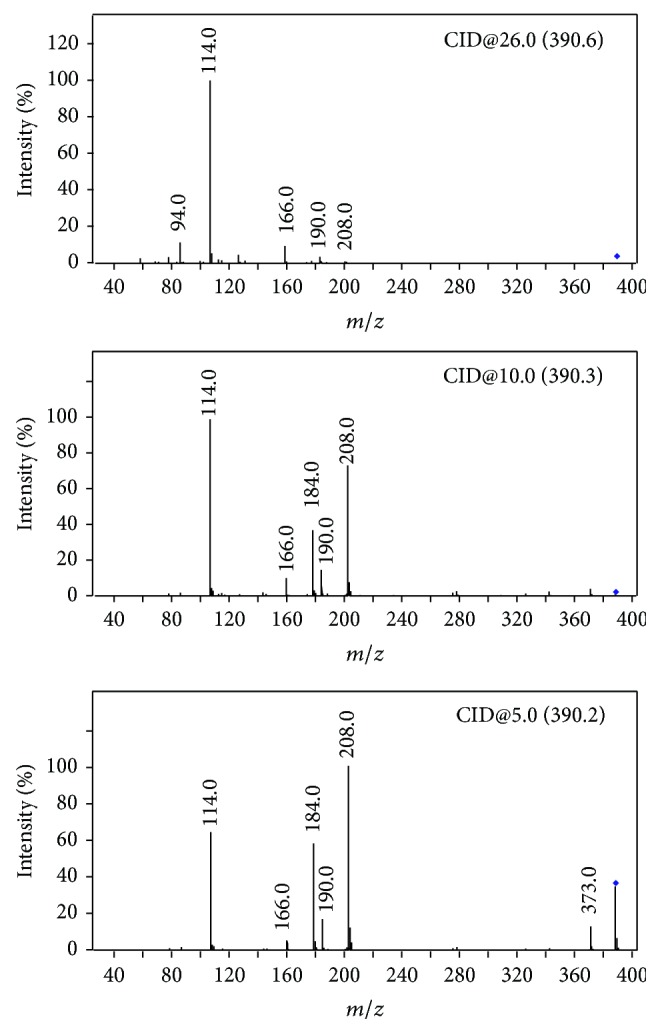
CID spectra of cefprozil activated under different activating voltage. The spectra were obtained with a Q-TOF. The activating voltages were 26 volt, 10 volt, and 5 volt, respectively.

Cleavage of *β*-lactam ring was the main, common, and primary fragmentation observed in the low energy activated CID spectrum of most *β*-lactams [20, 21]. Cefixime, cefdinir, cefaclor, and cefprozil exhibit this characteristic fragmentation under the condition of this job (Figures S-2 A, B, and C). However, there are exceptions sometimes. High yield of *β*-lactam ring cleavage was not observed to be in low energy activated CID spectrum of amoxicillin (Figure S-2 D) under the condition of this experiment, while it was prominent in a CID spectrum obtained under a relatively higher activating voltage [22]. CID spectrum of amoxicillin obtained under the condition of this experiment is dominated by the fragment* m/z* 349 [M+H–NH_3_]^+^ which is proposed to originate from the cleavage of C-N bond of the 4-hydroxybenzyl amine. This fragmentation channel has been also observed in the CID spectrum of cefprozil (*m/z* 373) [23] and cefadroxil (*m/z* 347) [15, 24]. It is probably driven by the production of a stable 4-hydroxybenzyl cation and a volatile molecule of NH_3_. The hydroxyl group localized on the paraposition is important to the activity of the amino group. The positive charge is stabilized by the conjugation effects provided by the hydroxyl group. Very low yield of this fragmentation (*m/z* 352) was observed in CID spectrum of cefaclor for the absence of the hydroxyl group. In fact, this amino group of cefprozil and amoxicillin is more prone to fragmentation than the *β*-lactam ring.  Figure 4 shows the CID spectra of cefprozil activated under different activating voltage. Fragment at* m/z* 373 was observed in the CID spectra obtained under very low activating voltage. This cation dissociates to form fragments that correspond to *β*-lactam ring cleavage (*m/z* 208,* m/z* 190, and* m/z* 184) under relatively high activating voltage. The cations at* m/z* 190 and* m/z* 184 were a set of complementary fragment ions originating from cleavage of the *β*-lactam ring of the deaminated fragment of* m/z* 373 in the CID spectrum of cefprozil. High yield of *β*-lactam ring cleavage reaction may also be achieved in the CID spectrum of amoxillin under this condition [15, 24].

**Figure 5:**
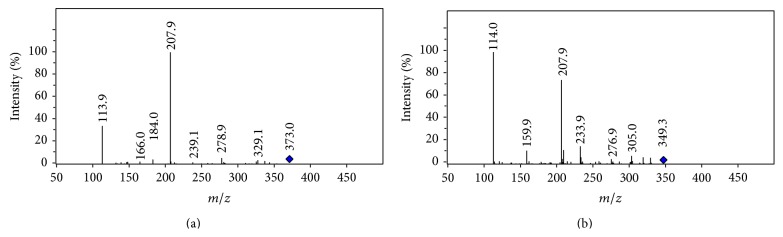
Represent CID spectra of deaminated daughter ion of cefprozil (a) and amoxillin (b). The spectra were obtained with the ion trap. The resonance voltages were both 1 volt, and the activating times were both 20 millisecond. The activating widths were both 10 amu.

Interestingly, the fragment that represents the cleavage of *β*-lactam ring was not observed at* m/z* 207 but at* m/z* 208 in both CID spectra of amoxillin and cefprozil. This fragment was proved to be formed from rearranged fragment at either* m/z* 373 or* m/z* 349 by the MS^3^ experiment of cefprozil and amoxicillin (Figure 5). A hydroxyl group has transformed to the benzyl carbon before its dissociation (Scheme 2). The formation of the fragment at* m/z* 114 from extensive elimination of a phenol from the cation* m/z* 208 was also confirmed by MS^3^ experiment (data not shown).

The production of fragments at* m/z* 208 and 114 is also observed in CID spectrum of cefadroxil [15, 24]. The reason for which fragment* m/z* 190 was not observed in Figure 5 but Figure 4 is that Figure 5 was obtained under relatively low activating voltage (approximately 1 v). It displays a prominent fragment originating from *β*-lactam ring cleavage of the rearrangement deaminated cation. Structural rearrangements are in competition with the fragmentations [20]. Low activating energy is advantageous for the structural rearrangement of the cation during the process of CID.

Cefuroxime is another exception of *β*-lactam profile (Figure S-4) in this job. Because there are unstable substituent groups structurally, protonated cefuroxime was not observed in its all scan spectrum (Figure S-4 A). Ammonium adduct [M+NH_4_]^+^ and sodium adduct [M+Na]^+^ were observed in a considerable abundance. The daughter ions observed in all scan spectra indicated that the molecule was so unstable that the dissociation occurred in the ion source (source CID). There is not any fragment, which corresponds to *β*-lactam ring cleavage of *β*-lactam ring, observed in the MS^2^, MS^3^, and MS^4^ CID spectra of cefuroxime ammonium adduct (Figures S-4 B, C, and D). However, it was reported that high yield of *β*-lactam ring cleavage has been observed under optimized collision condition for this kind of compounds [25]. Considerable yield of fragment represents *β*-lactam ring cleavage was observed in the CID spectrum of cefuroxime in negative mode (Figures S-4 E, F, G, and H).

Above all, *β*-lactam ring cleavage is generally prominent fragmentation in the low energy CID spectra of *β*-lactam antibiotics.

### 3.4. CID Spectra of Cefixime and Ring-Opened Cefixime Activated under High Resonance Voltage

**Figure 6:**
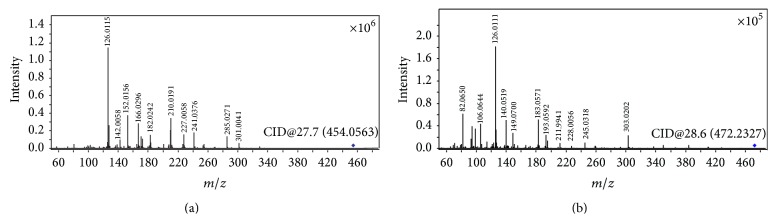
High energy activated CID spectra of cefixime (a) and ring-opened cefixime (b). The spectra were obtained with a Q-TOF under the mode of auto MS/MS. The activating voltages were 27.7 volt and 28.6 volt, respectively.

Figure 6 shows the high energy activated CID spectra of cefixime and ring-opened cefixime. Both spectra exhibit similar appearance, for example, the same base peak (*m/z* 126), almost the same* m/z* value and abundance for all the corresponding fragments. In contrast, low energy activated CID spectrum of impurities with *β*-lactam ring can be rapidly recognized from those of lactam ring-opened impurities. The similar high energy activated CID spectral profiles have been observed for all the *β*-lactam antibiotics and their impurities.

The profile of the CID spectra of *β*-lactams, which displays a prominent fragment originating from the cleavage of its *β*-lactam ring, was named as *β*-lactam profile in this job. Accordingly, the profile of ring-opened impurities of *β*-lactam antibiotics, which is dominated by the fragments formed from elimination of small molecules, was called ring-opened lactam profile. For most of *β*-lactam antibiotics, characteristic CID can be obtained with a resonance ion trap whose activating voltage is typically lower than 2 volts. For those *β*-lactam antibiotics with unstable substituent group, the characteristic CID spectra can be obtained under relatively higher activating voltage, typically activated under 5–10 volts with a hybridized mass spectrometry with fast activating MS/MS function, for example, triple quadrupole mass spectrometry or Q-TOF. The CID spectra of *β*-lactam antibiotics obtained under high activating energy did not display these characteristic profiles. These characteristics can be used to differentiate ring-opened *β*-lactam impurities from those with *β*-lactam ring.

### 3.5. Identification of Impurities in Cefixime

#### 3.5.1. Identification of Compound 1

**Scheme 4:**
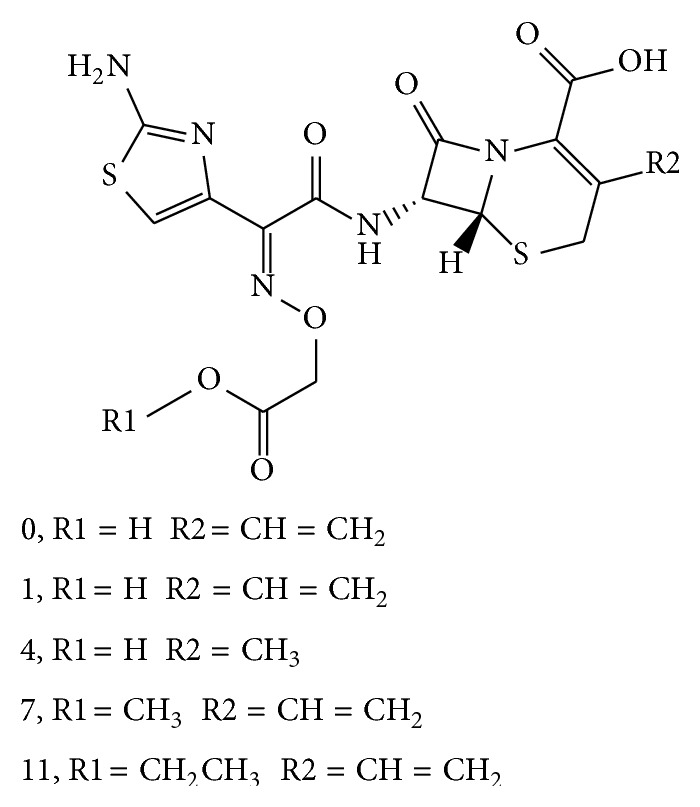
Structures of cefixime and impurities with *β*-lactam ring.

There are thirteen impurities detected in both cefixime capsulars (Figure 2) analyzed in this job. Most of them appeared to be stereoisomeric pairs. The molecular weight of compound 1 is the same as cefixime. And its CID spectrum displays the same appearance as that of cefixime (Figure S-1 A). The fragment ion* m/z* 285 formed from cleavage of lactam ring suggested that it was a *β*-lactam. Compound 1 was proposed to be an isomer of cefixime on the basis of these observations. It was identified as cefixime impurity C or impurity D (Scheme 4) in accordance with cefixime impurities reported in the literature [25].

#### 3.5.2. Identification of Compounds 4, 7, and 11

There was not isomer of compounds 4, 7, and 11 observed in cefixime. The display of *β*-lactam profile of their CID spectra (Figures S-1 B, C, and D) indicated that there was thiazine lactam ring in each compound. The pseudomolecular ion of compound 4 is at* m/z* 442 [M+H]^+^, which is 12 amu different from that of cefixime, which suggested that its molecular weight was 441. The fragment at* m/z* 285, as well as the fragments at* m/z* 241, 182, 166, and 126, suggested that the side chain of compound 4 was the same as cefixime. The fragment ion* m/z* 329 indicated there was no other substituent group localized on the lactam ring. These observations indicated that the smaller molecular weight was due to the substituent group localized on the thiazine ring of compound 4, which is 12 amu less than a vinyl group. Based on these observations, compound 4 was identified as cefixime impurity E [25] (Scheme 4).

Compound 7 displays the same fragmentation channels as cefixime. The fragments at* m/z* 299 and* m/z* 255 originate from the same fragmentation channel as* m/z* 285 and* m/z* 241 in the CID spectrum of cefixime (Scheme S-1). The fragment ion* m/z* 343 was formed from the same mechanism as* m/z* 329 (Scheme S-1). These fragments indicated that the side chain of compound 7 was different from which of cefixime. Display of fragment at* m/z* 210,* m/z* 182, and* m/z* 166 in the CID spectrum of compound 7, which originates from the same fragmentation channel with those of cefixime (Scheme 1 R-2), indicated that the substituent group on the oxime was different from that of cefixime. Compound 7 was identified as (6R,7R)-7-[[(Z)-2-(2-aminothiazol-4-yl)-2-[(2-methoxy-2-oxoethoxy)imino]acetyl]amino]-3-ethenyl-8-oxo-5-thia-1-azabicyclo[4.2.0]oct-2-ene-2-carboxylic acid (Scheme 4), which is an intermediate product from cefixime synthesis. This impurity was not reported in the literature. Similarly, compound 11 was identified to be cefixime impurity F (Scheme 4) [25].

#### 3.5.3. Identification of Compounds 5, 6, 8, 9, 10, 12, and 13

**Scheme 5:**
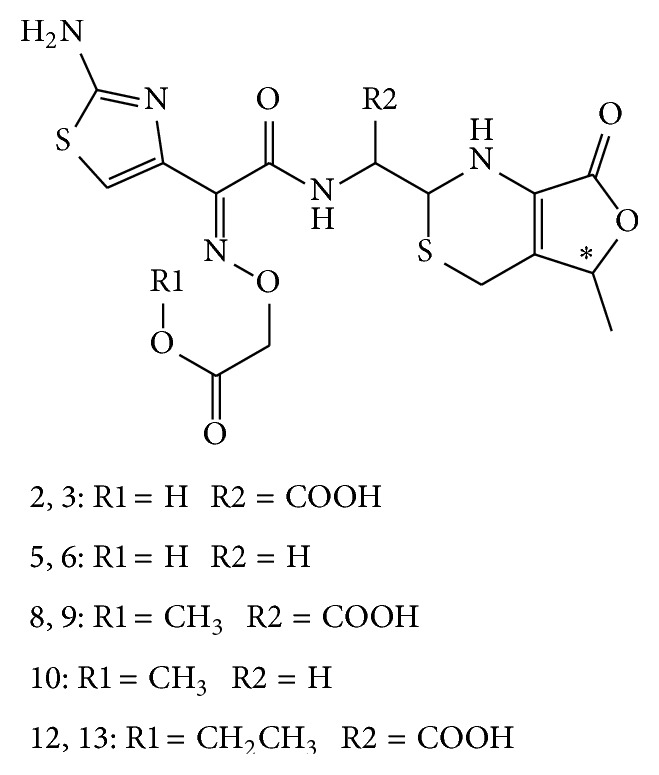
Structure of ring-opened impurities of cefixime.

All of low energy activated CID spectra of compounds 5, 6, 8, 9, 10, 12, and 13 display profiles of ring-opened *β*-lactam (Figure S-3). Compound 5 and compound 6 are isomers. Their protonated molecule (*m/z* 428) indicated its molecular weight was 44 amu smaller than cefixime impurity A (Figure S-3 B). All daughter fragments were observed to be the same as displayed in the CID spectra of impurity A. Compounds 5 and 6 were identified as the stereoisomers of decarboxylated impurity A, 2-[[[(E)-1-(2-aminothiazol-4-yl)-2-[[[(2R,5R/S)-5-methyl-7-oxo-1,2,5,7-tetrahydro-4H-furo[3,4-d][1,3]thiazin-2-yl]methyl]amino]-2-oxoethylidene]amino]oxy]acetic acid based on above observations (Scheme 5).

Compounds 8 and 9 are stereoisomers. The observation of pseudomolecular ion at* m/z* 486 indicated their molecular weight was 485, which is 18 amu more than compound 7 (Figure S-3 C). The appearances of their CID spectra are both in ring-opened cefixime profile. The observation of fragment at* m/z* 337 suggested that the structure of either compound 8 or compound 9 was the same as compound 3, except for the substituent group localized on the oxime. These observations suggested that they are lactam ring-opened product of compound 7. Compounds 8 and 9 were identified as 2-[[(E)-2-(2-aminothiazol-4-yl)-2-[(2-methoxy-2-oxoethoxy)imino]acetyl]amino]-2-[(2R,5R/S)-5-methyl-7-oxo-1,2,5,7-tetrahydro-4H-furo[3,4-d][1,3]thiazin-2-yl]acetic acid (Scheme 5) based on these data.

The pseudomolecular ion of compound 10 was observed at* m/z* 442. Its CID spectrum (Figure S-3 D) also appears in a ring-opened cefixime profile. All the daughter ions were observed to be the same as compounds 8 and 9. These observations suggested it was a decarboxylated product of compounds 8 or 9. Based on these data, compound 10 was identified to be 2-[[(E)-2-(2-aminothiazol-4-yl)-2-[(2-methoxy-2-oxoethoxy)imino]acetyl]amino]methyl-5-methyl-4,5-dihydro-1H-furo[3,4-d][1,3]thiazin-7(2H)-one (Scheme 5).

Compounds 12 and 13 are also stereoisomers. Their CID spectra (Figure S-3 E) display a ring-opened cefixime profile. The pseudomolecular ion at* m/z* 500, which is 18 amu more than cefixime impurity F, suggested they were lactam ring-opened product of cefixime impurity F. Based on these observations, compounds 12 and 13 were identified as 2-[[(E)-2-(2-aminothiazol-4-yl)-2-[(2-ethoxy-2-oxoethoxy)imino]acetyl]amino]-2-[(2R,5R/S)-5-methyl-7-oxo-1,2,5,7-tetrahydro-4H-furo[3,4-d][1,3]thiazin-2-yl]acetic acid (Scheme 5).

Among these impurities, the molecular weight of both compound 4 and compound 10 is 441 amu. However, the CID spectrum of compound 4 displays a *β*-lactam CID spectral profile, in contrast, which of compound 10 displays in ring-opened *β*-lactam CID spectral profile. They are successfully differentiated by their characteristic CID spectral profiles.

### 3.6. CID Characteristics of Cefdinir and Its Impurities

#### 3.6.1. Diagnostic Fragmentations of Cefdinir

As the same as the CID spectrum appearance of cefixime, low energy activated CID spectrum of cefdinir (Figure S-2 A) is typically in *β*-lactams CID spectral profile. The fragment* m/z* 227, formed from the cleavage of *β*-lactam ring (Scheme S-6), may sever as diagnostic fragment to determine the localization of substituent groups of unknown impurities. The fragment* m/z* 183 is proposed to be formed from rearrangement of the said chain rather than from thiazine lactam [25] (Scheme S-6 R-2) because it was also observed in the CID spectrum of cefdinir impurity C (Mw, 383 Figure S-5). This fragmentation, as well as the fragment* m/z* 126, was also used to determine the localization of substituent groups.

#### 3.6.2. Diagnostic Fragmentations of Ring-Opened Cefdinir

**Scheme 6:**
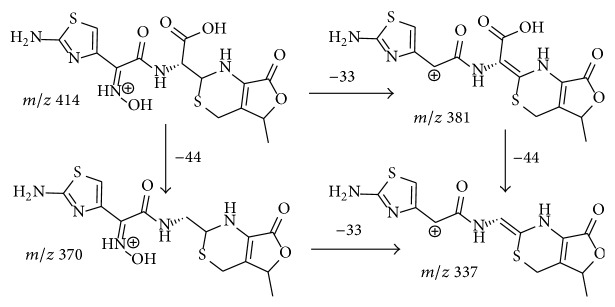
Proposed pathway for the formation of fragment at* m/z* 337.

**Figure 7:**
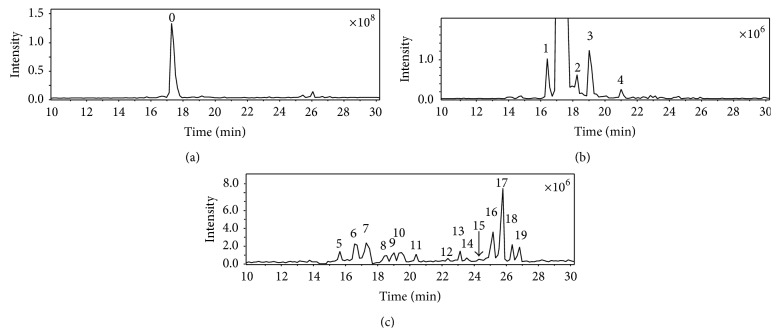
Representative chromatogram of cefdinir capsule and its impurities. 0 represents the major constituent.

There are four compounds whose molecular weight was found to be 413 in the capsule of cefdinir (Figure 7, compounds 6, 7, 8, and 9). The CID spectra of all these compounds were also observed to be in an abovementioned profile of ring-opened *β*-lactam impurities, displaying prominent fragments originate from dehydrate and decarboxylation (Figure S-2 E and Figure S-6). Based on the these observations, compounds 6, 7, 8, and 9 were all identified as ring-opened cefdinir, which is named as cefdinir related compound A in the United States pharmacopeia [26]. Considerable yield of fragments formed from a neutral loss at 33 amu (*m/z* 381 [M+H-33]^+^, 337 [370-33]^+^) was observed in CID spectra of compound 6, compound 7, and also several other ring-opened impurities. This neutral molecule was proposed to be hydroxylamine (NH_2_OH) which originates from the rearrangement of the side chain (Scheme 6). The force for this fragmentation is attributed to the formation of very stable products. The fragmentation of loss hydroxylamine could even be observed in the CID spectra of cefdinir (*m/z* 363) and impurity C of cefdinir (*m/z* 351), though in low abundance (approximately 2%, Scheme S-6 R-3) which is attributed to the structural rigidity of the *β*-lactam ring. Hydroxylamine is better leaving group than 2-(aminooxy)acetic acid, the yields of fragment at* m/z* 337 in the CID spectra of ring-opened impurities of cefdinir are much higher than those in the spectra of cefixime related impurities (Figure S-3) thereof. This fragmentation (loss hydroxylamine) is a very important diagnostic character for determining the location of the substituent groups of ring-opened impurities related to cefdinir. In addition, the abundances of the fragment at* m/z* 337 and* m/z* 381 are quite different in the CID spectra of ring-opened cefdinir (compounds 6, 7, 8, and 9). This observation suggested that the configuration of C-2 of thiazin ring was responsible the yield of fragments formed from this rearrangement. Therefore, the relative abundances of the daughter ion originated from this fragmentation are hoped to be used as a diagnostic character to determine the configuration of C-2 of ring-opened impurities of cefdinir. Unfortunately, we failed to collect proper references to determine the configuration of these compounds.

The representative chromatograms of cefdinir and its impurities are shown in Figure 7. Among these impurities, there were five impurities (compounds 1–5), whose CID spectra have shown *β*-lactams appearance, and fourteen impurities (compounds 6–19), whose CID spectra have shown ring-opened *β*-lactams appearance. The identification of all these impurities was described in the supporting data.

### 3.7. Diagnostic Fragmentations of Cefaclor and Ring-Opened Cefaclor

#### 3.7.1. Fragmentation Pattern of Cefaclor

**Scheme 7:**
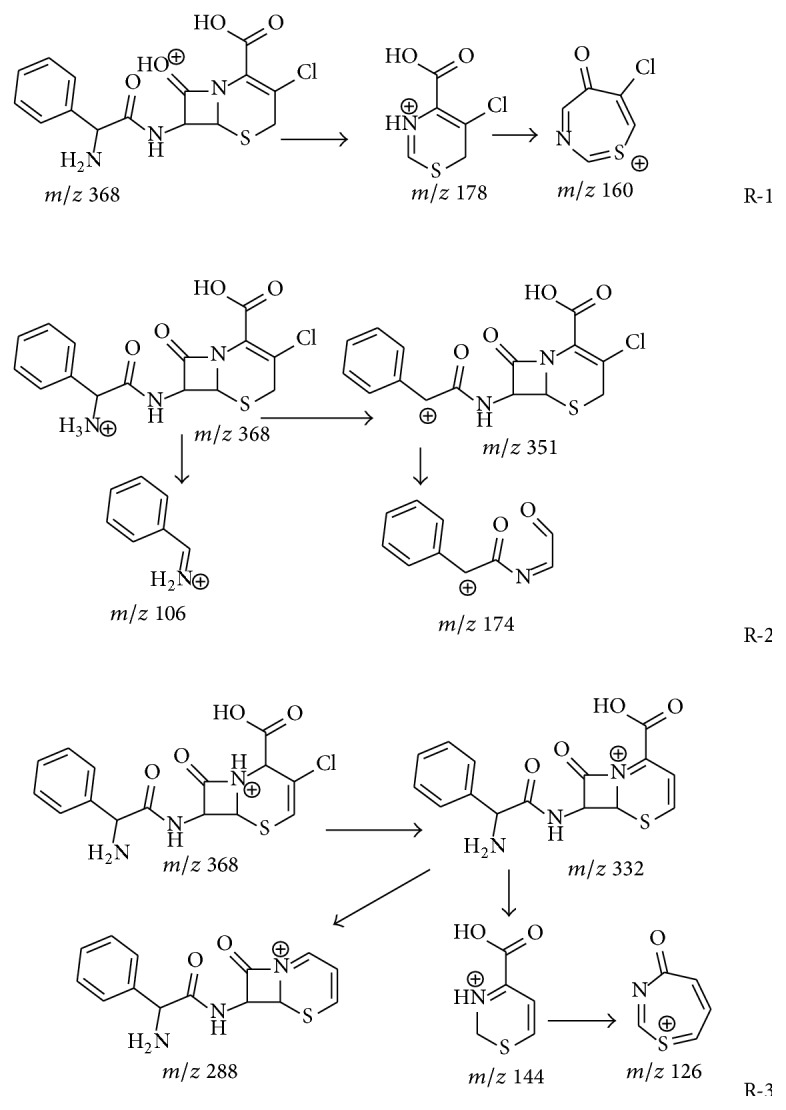
Proposed fragmentation pattern for cefaclor and delta-3 cefaclor.

**Figure 8:**
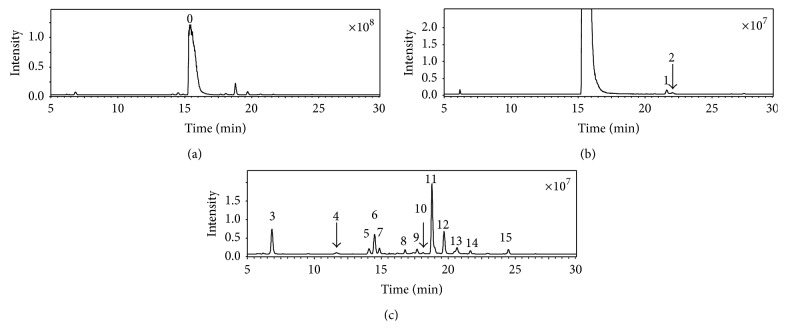
Representative chromatograms of cefaclor capsule and its impurities. 0 represents the major constituent.

**Figure 9:**
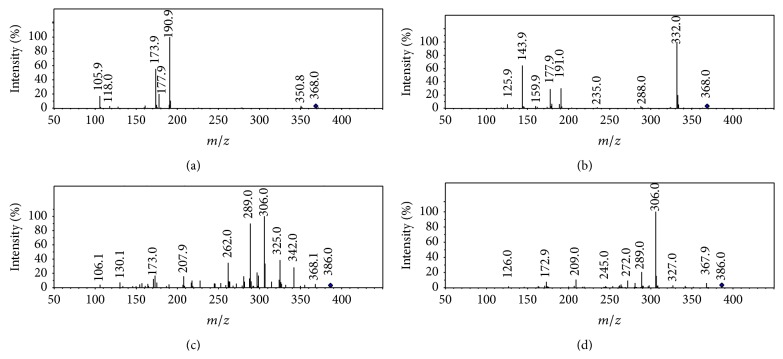
Low energy activated CID spectra of cefaclor (a), delta-3 cefaclor (b), and corresponding ring-opened compounds ((c) and (d)).

Figure 8 shows the representative chromatograms of cefaclor and its impurities. As similar to that of cefixime and cefdinir, the CID spectrum of cefaclor displays a profile of *β*-lactam CID spectrum (Figure 9(a)). The fragments formed from cleavage of lactam ring dominate the spectrum. The fragments originate from the fragmentation channel (Scheme 7) of ions at* m/z* 191,* m/z* 174,* m/z* 118, and* m/z* 106 may be used to determine the structure of the side chain, while those produced from the fragmentation channel of ion* m/z* 178 may be used to determine the substituent groups localized on thiazine ring.

Both protonated compound 1 and protonated compound 2 were observed at* m/z* 368 (Figure 9(b)), and both exhibit high yield fragments originating from cleavage of *β*-lactam ring. The CID spectra of compounds 1 and 2 are dominated by the fragment formed from elimination of hydrogen chloride, which is absent from the CID spectrum of cefaclor. The different yields of the elimination of hydrogen chloride is attributed to that the hydrogen localized on the C-2 of compounds 1 or 2 (Scheme 7 R-3) is much more active than that on the C-4 of cefaclor. Double bond at delta-3 is advantageous to hydrogen chloride elimination. This fragmentation may be used to determine the localization of the double bond on the thiazine ring thereof (Scheme 7 R-3). The exhibition of fragments at* m/z* 191 indicated the side chains of both compounds 1 and 2 were the same as that of cefaclor. Fragments at* m/z* 178,* m/z* 160, and* m/z* 144 represent the structure of thiazine ring. Compounds 1 and 2 were identified as delta-3-cefaclor (impurity D of cefaclor) based on these observations (Scheme S-10 A).

#### 3.7.2. Fragmentation Pattern of Ring-Opened Cefaclor

The recognizing of ring-opened cefaclor is based on their molecular weight. Among these impurities whose molecular weight is 18 amu more than that of cefaclor was possibly ring-opened cefaclor. There are two compounds (compounds 9 and 10) found in the HPLC MS data of cefaclor capsule. CID spectra of both compounds 9 and 10 display a similar profile as the ring-opened *β*-lactams (Figure S-2 F, Figures 9(c) and 9(d)) identified in cefixime and cefdinir. CID spectrum of compound 9 displays high yields of fragments formed from elimination of small molecules, for example, H_2_O, CO_2_, NH_3_, and HCl (*m/z* 306, 100%). These observations confirmed the structure of ring-opened cefaclor. The CID spectrum of compound 10 displays very high yield of fragment at* m/z* 306 (100%) but lower yield of fragments at* m/z* 342 [M+H-44]^+^,* m/z* 325 [342-17]^+^, and* m/z* 262 [306-44]^+^. As described, localization of the double bond on the thiazine ring affects the yields of HCl (Scheme 7). The double bond of compound 9 was suggested to locate between C-4 and C-5 (delta-4), which of compound 10 locates between C-5 and C-6 (delta-5), based on these observations. Double bond at delta-5 is advantageous to eliminate the chloride at C-5, while delta-4 double bond is propitious to decarboxylation at C-4 thereof. The low yield of fragment which originates from elimination of CO_2_ in the CID spectrum of compound 10 was suggested to be resulting from the delta-5 double bond on the thiazine ring. Based on these data, compound 10 was identified to be the delta-5-isomer of compound 9. Therefore, compound 9 was identified as 2-((R)-2-amino-2-phenylacetamido)-2-(5-chloro-4-(carboxy)-3,6-dihydro-2H-1,3-thiazin-2-yl)acetic acid, compound 10 was identified as 2-((R)-2-amino-2-phenylacetamido)-2-(4R/S-5-chloro-4-(carboxy)-3,4-dihydro-2H-1,3-thiazin-2-yl) acetic acid (Scheme S-11).

There were fifteen impurities detected in the cefaclor capsular (Figure 8). Among these impurities, there were four impurities (compounds 1, 2, 14, and 15), whose CID spectra have shown *β*-lactams appearance, and eleven impurities (compound 3–12), whose CID spectra have shown ring-opened *β*-lactams appearance. The identification of all these impurities was described in the supporting data.

## 4. Conclusions 

CID spectra activated under low resonance voltage of *β*-lactams are generally dominated by fragments originate from *β*-lactam ring cleavage. In contrast, those of ring-opened *β*-lactams are dominated by fragments formed from elimination of small molecules, for example, H_2_O and CO_2_. These characters, called *β*-lactam profile or ring-opened lactam profile, respectively, were successfully used as diagnostic characteristics to differentiate *β*-lactams from ring-opened *β*-lactams. Overall forty-five impurities were identified from cefixime, cefdinir, and cefaclor. However, these characteristic fragments were extensively dissociated under high activating energy CID, and *β*-lactams and ring-opened *β*-lactams were observed to exhibit similar appearances of CID spectra under this condition.

## Supplementary Material

Supplementary Material: For the sake of better understanding the different CID profiles of beta-lactams and their ring-opened impurities, additional information about the CID reactions and CID spectra of some compound, as well as the identification of impurities in cefdinir and cefaclor, were provided as “Supplementary Material”.
