# Supplementary material for: Mass Spectral Profile for Rapid Differentiating Beta-Lactams from Their Ring-Opened Impurities
Source: Biomed Res Int. 2015 May 18;2015:697958. doi: 10.1155/2015/697958 (PMC4450268; doi:10.1155/2015/697958)
Supplement: Supplementary file 1 — Supplementary Material: For the sake of better understanding the different CID profiles of beta-lactams and their ring-opened impurities, additional information about the CID reactions and CID spectra of some compound, as well as the identification of impurities in cefdinir and cefaclor, were provided as “Supplementary Material”. [file 697958.f1.pdf]

Supporting data

Mass spectral profile for rapid differentiating beta-lactams from their ring-opened impurities

Hecheng Wang[1,2], Haiwei Huang[3], Jin Cao[3], Dehua Chui[2], Shengyuan Xiao[1]

[1] School of Life Science, Beijing Institute of Technology, Beijing 100081, China
[2] Neuroscience Research Institute, Health Science Center, Peking University, Beijing 100191, China
[3] National Institutes for Food and Drug Control, Beijing 100050, China

Correspondence should be addressed to Shengyuan Xiao; shyxiao@gmail.com

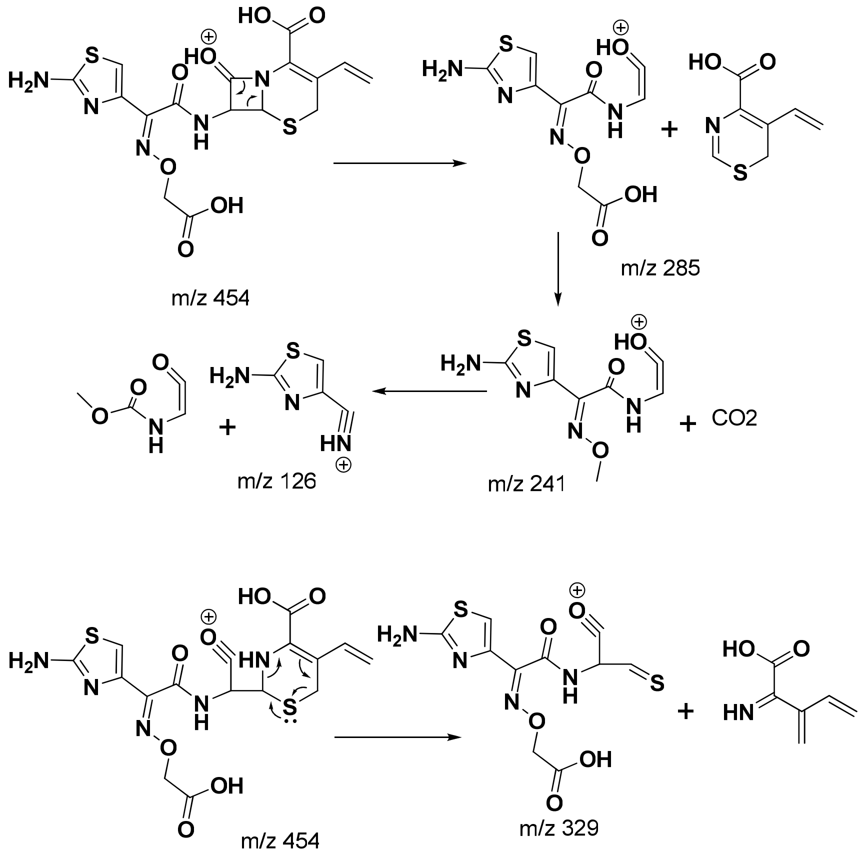

Scheme S-1: Proposed fragmentation pattern for fragments at m/z 329, m/z 241 and m/z 126.

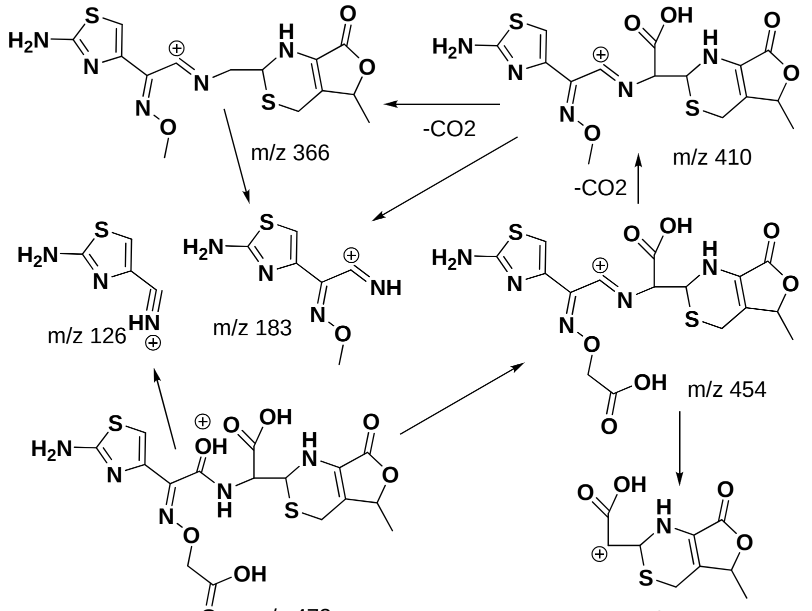

Scheme S-2: Proposed fragmentation pattern of ring-opened cefixime activated under low activating voltage.

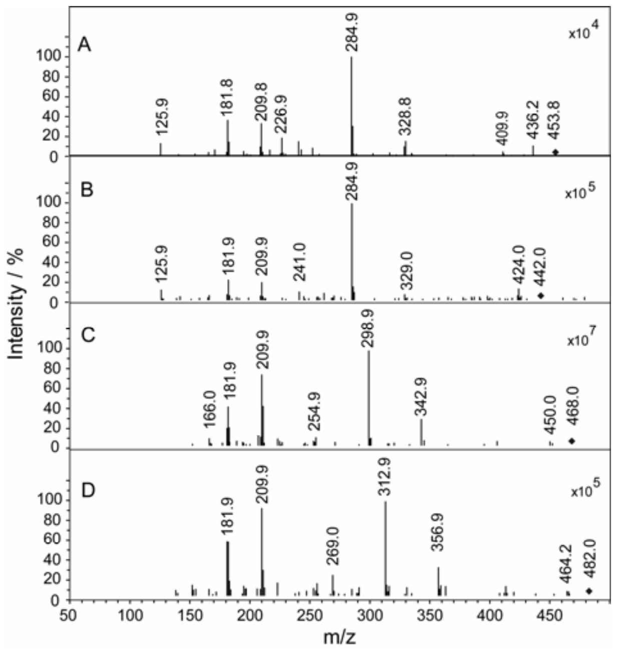

Figure S-1: Low energy activated CID spectra of impurities with b-lactam ring in cefixime capsule. A, compound 1; B, compound 2; C, compound 7; D, compound 11.

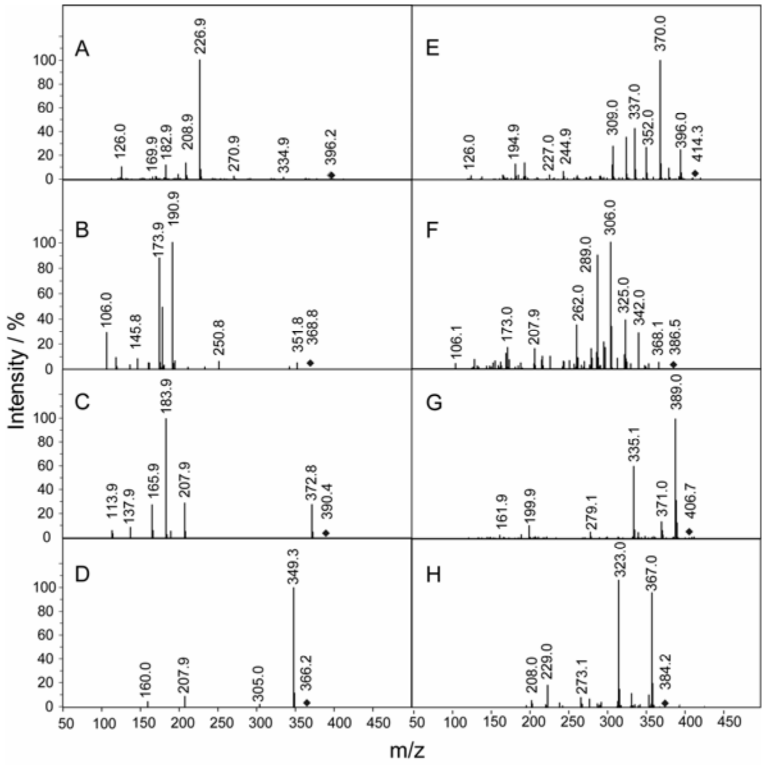

Figure S-2: Low energy activated CID spectra of b-lactam antibiotics and corresponding ring-opened impurities. A, cefdinir; B, cefaclor; C, cefprozil; D, amoxicillin; E, ring-opened cefdinir; F, ring-opened cefaclor; G, ring-opened cefprozil; E, ring-opened amoxillin.

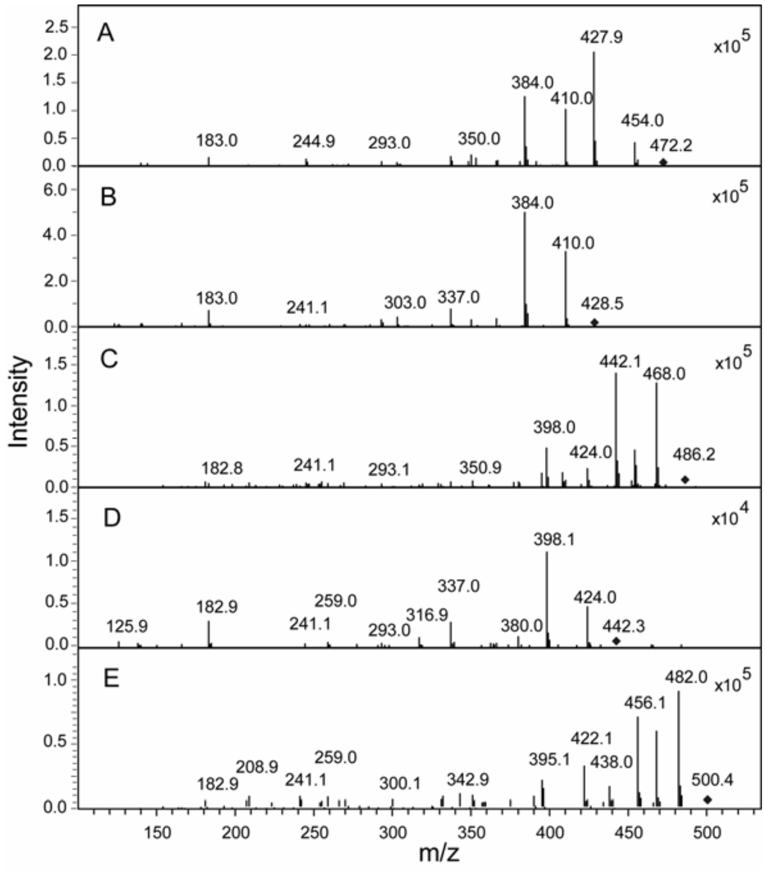

Figure S-3: Low energy activated CID spectra of ring-opened detected in cefixime capsule. A, compound 2 and 3; B, compound 5 and 6; C, compound 8 and 9; D, compound 10; E, compound 12 and 13.

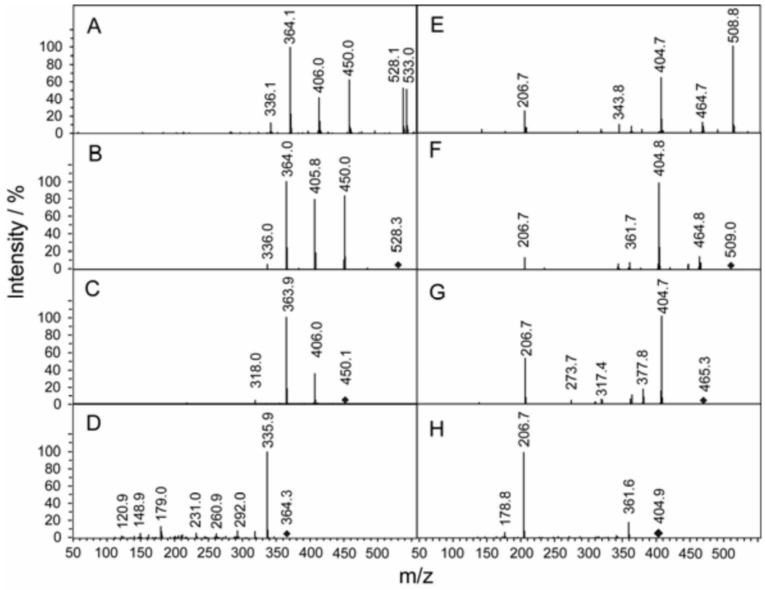

Figure S-4: Low energy activated CID spectra of cefuroxime. A – D, CID spectra obtained under positive mode; A,

MS^2; B – D, MS^3; E-H, CID spectra obtained under negative mode; E, MS scan spectrum; F, MS^2; G and H, MS^3.

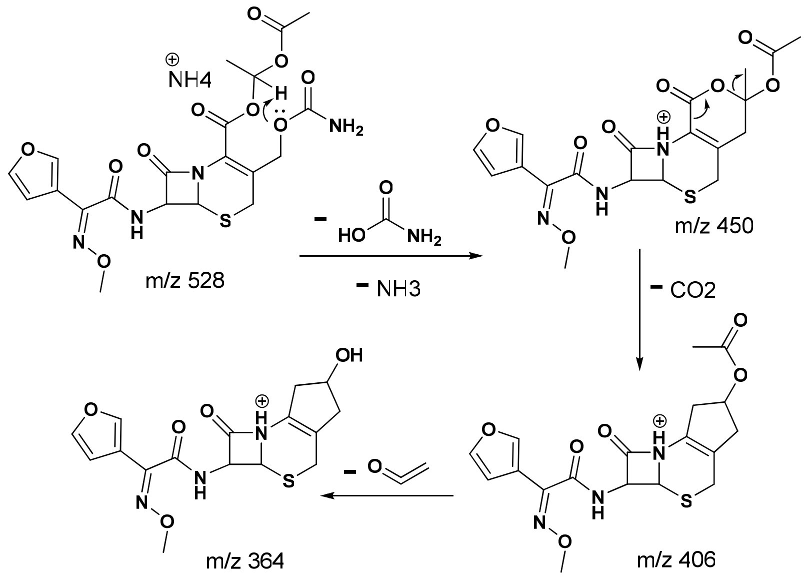

Scheme S-3: Proposed fragmentation pathway of cefuroxime.

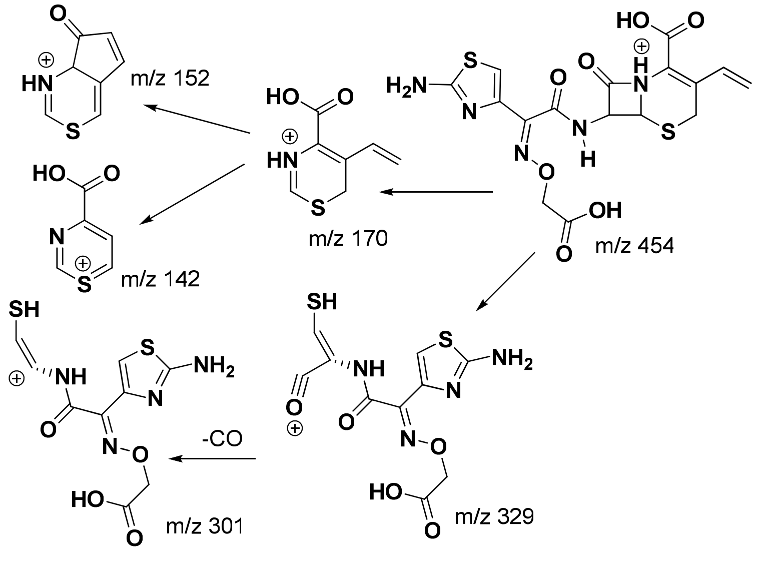

Scheme S-4: Proposed fragmentation pathway of cefixime under activating high energy.

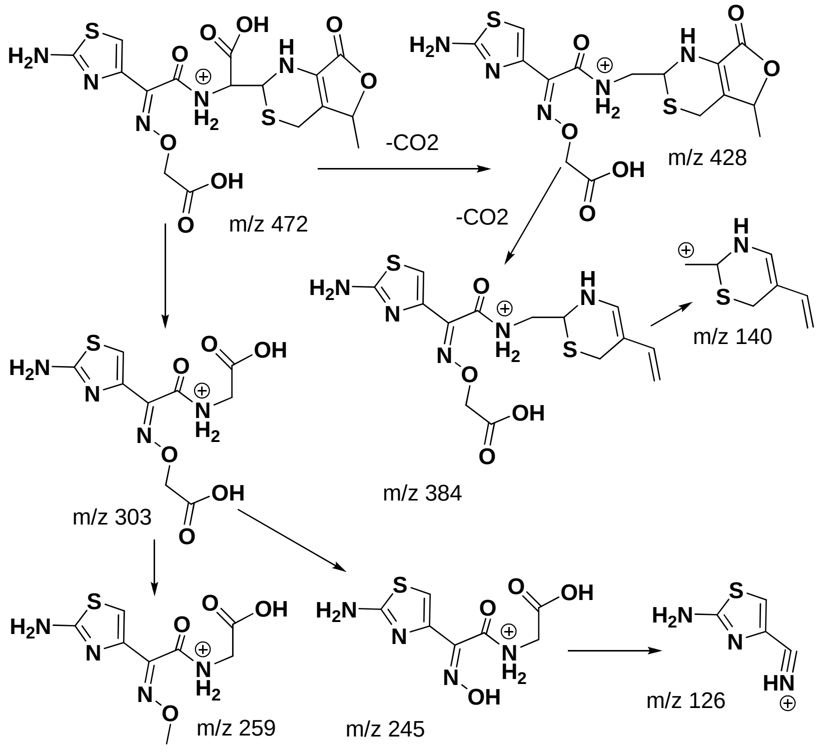

Scheme S-5: Proposed fragmentation pathway of ring-opened cefixime under high activating energy.

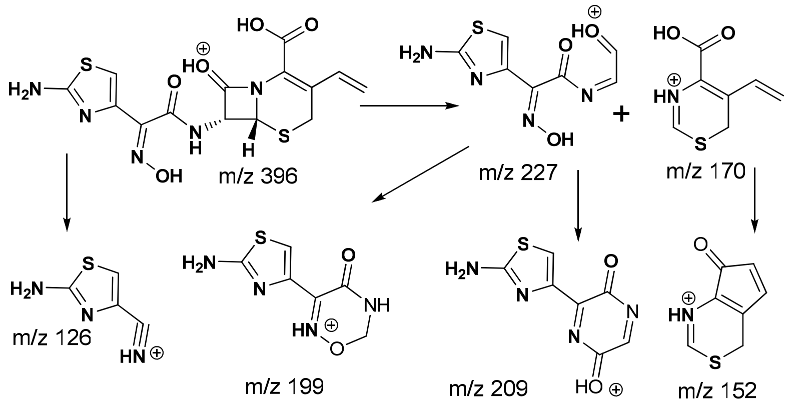

R-1

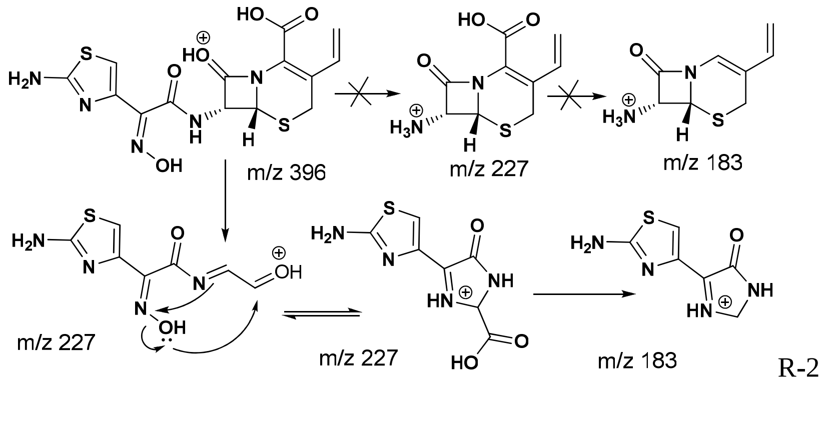

R-2

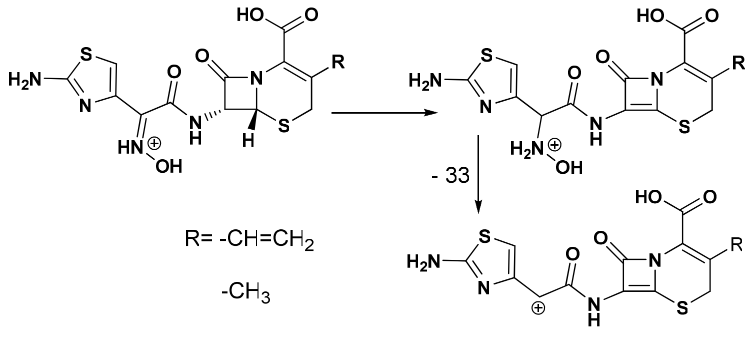

R-3

Scheme S-6: Proposed fragmentation pathway of cefdinir under low activating energy.

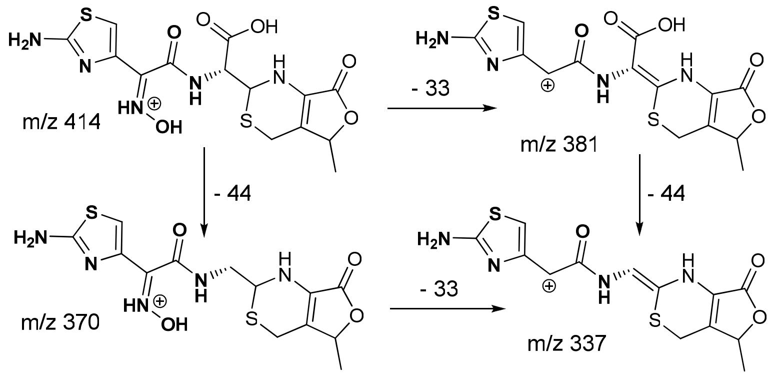

Scheme S-7: Proposed pathway of fragment at m/z 337 formation from ring-opened cefdinir under low activating energy.

Identification of impurities in cefdinir

The representative chromatograms of cefdinir and its impurities are shown in figure 7. Apart from the main constituent (Compound 0), there are four peaks observed in extracted ion chromatogram at *m/z* 396 (Figure 7-B). All display similar CID spectra as that of cefdinir. Compound 1`was proposed to be impurity E, cefdinir lactone product, according to the relative retention time and abundance (Scheme S-8). Compound 3 was identified similarly to be impurity G, cefdinir 7-epimer (Scheme S-8) . Compound 2 and 4 was suggested as unknown isomers.

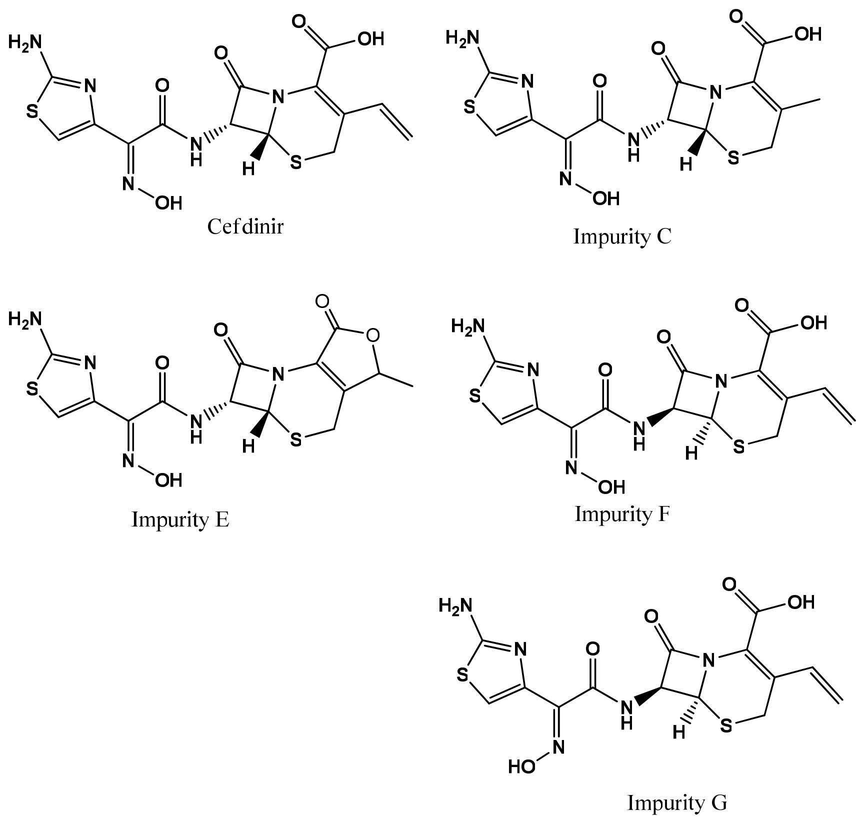

Scheme S-8: Structures of cefdinir and impurities with β-lactam ring.

The pseudo-molecular ion at *m/z* 384 indicates that the molecular weight of compound 5 was 383, which is the same as impurity C of cefdinir. Its CID spectrum is observed to be almost the same as which of cefdinir (Figure S-5). Compound 5 was identified as impurity C of cefdinir (Scheme S-8) based on these data.

Protonated molecules at *m/z* 370 suggested that compound 10 and 11 were decarboxylation products of ring-opened cefdinir (compound 6 or 8). Their CID spectra were also observed to be in a ring-opened β-lactam profile (Figure S-7). Different yields of fragments formed from loss of hydroxylamine were also observed in CID spectra of these compounds. Compound 10 and 11 were suggested to be impurity H of cefdinir (Scheme S-9) based on these data.

The pseudo-molecular ion of compound 12 and 13 were observed at *m/z* 428, which is 14 amu more than those of compound 6. The observation of fragment at *m/z* 395 (80% - 90%), as well as the fragments at *m/z* 126, suggested that the side chains of compound 12 and 13 were the same as that of compound 6, and different substituent group on the furanone. Based on these observation, compound 12 and 13 were tentatively identified as 2-[2-(2-Aminothiazol-4-yl)-2- (hydroxyimino)acetamido]-2-[5-ethyl-7-oxo-2,4,5,7-tetrahydro-1H-furo[3,4-d][1,3] thiazin-2-yl] acetic acid (Scheme S-9). Compound 14 – 19 were identified similarly to be

2-[2-(2-Aminothiazol-4-yl)-2-(hydroxyimino)acetamido]-2-[5-propyl-7-oxo-2,4,5,7-tetrahydro-1H-furo[3,4-d][1,3]thiazin-2-yl]acetic acid (Scheme S-9).

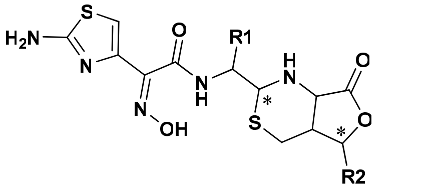

6 - 9 : R1 = COOH, R2 = CH_3
10, 11 : R1 = H , R2 = CH_3
12, 13 : R1 = COOH, R2 = CH_2CH_3
14 - 19: R1 = COOH, R2 = $CH_2CH_2CH_3$

Scheme S-9: Structures of the ring-opened impurities detected in the cefdinir capsule.

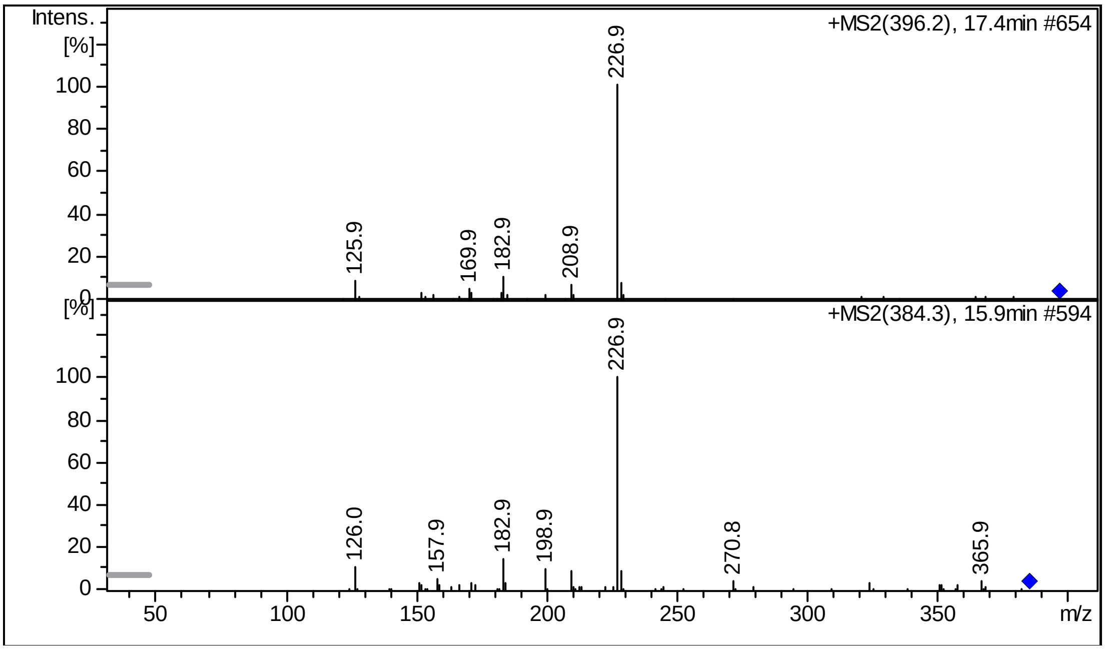

Figure S-5: Low energy activated CID spectrum of cefdinir impurities C (compound 5). Upper, cefdinir; Lower, Compound 5.

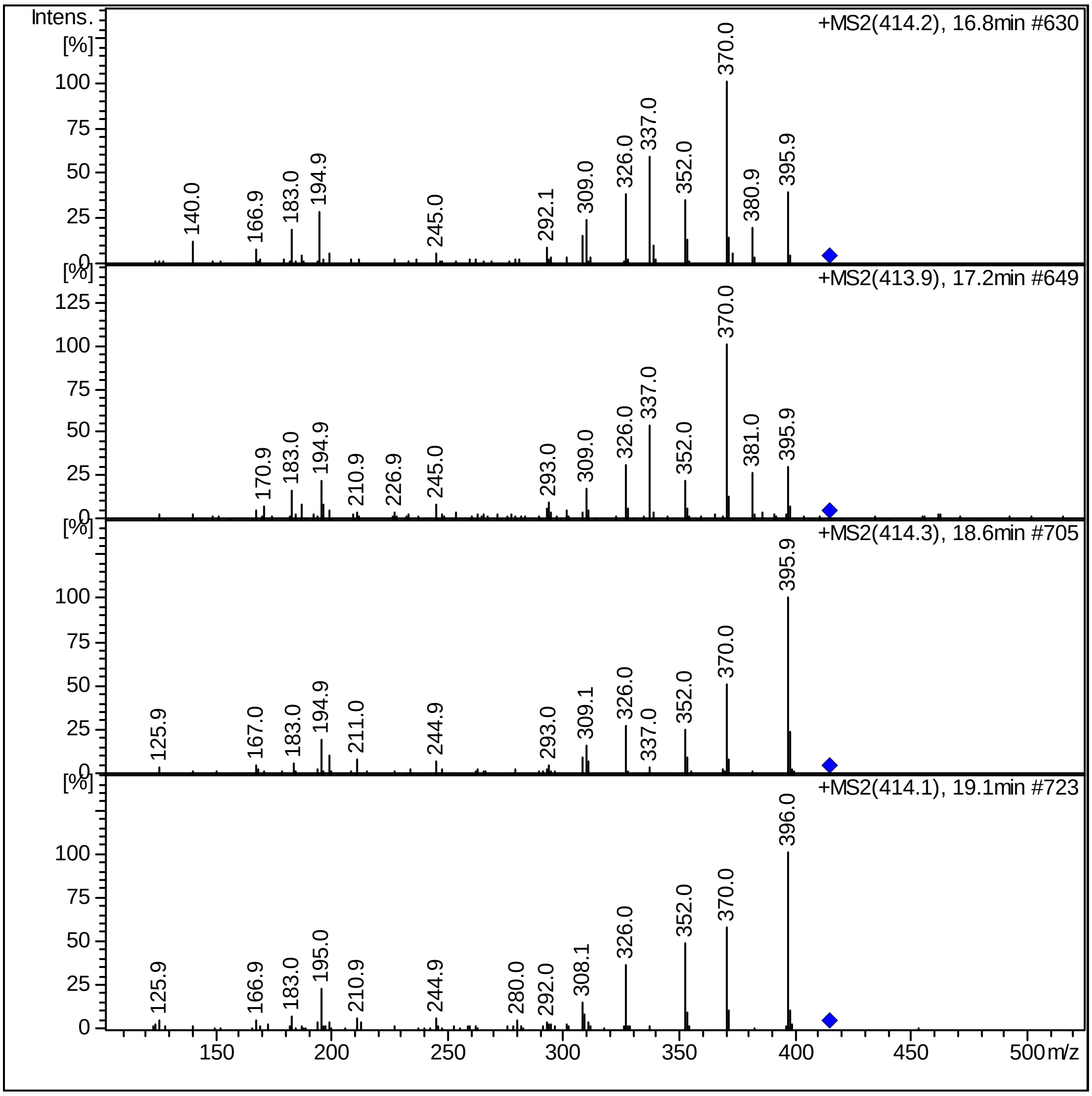

Figure S-6: Low energy CID spectra of cefdinir related compound A (compound 6, 7, 8 and 9)

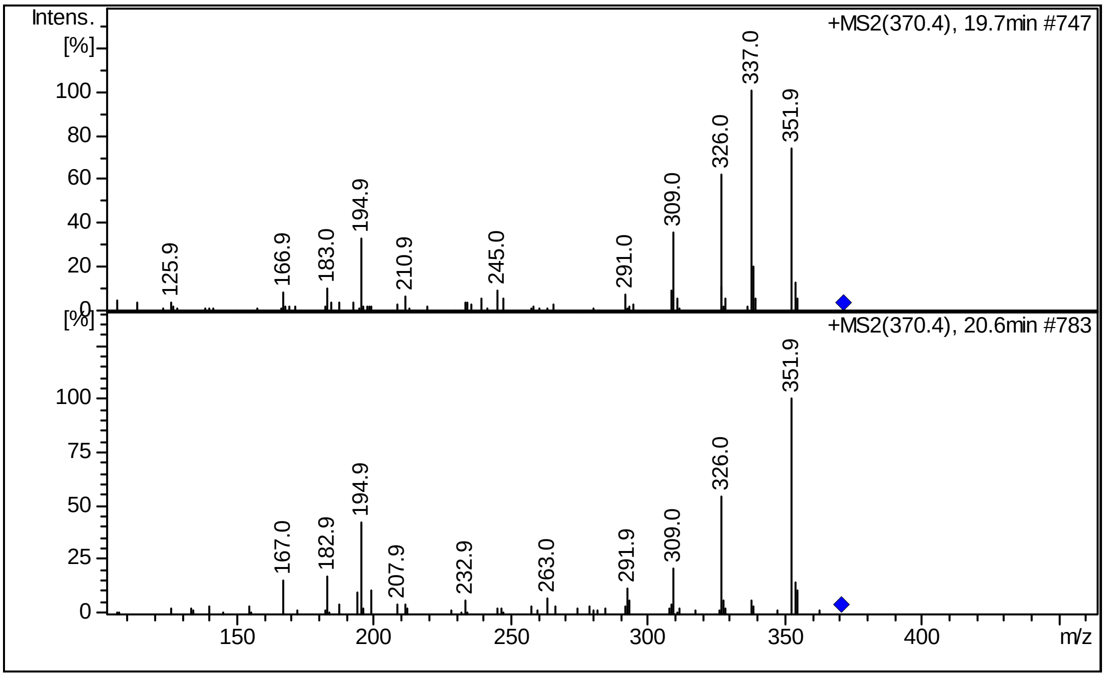

Figure S-7: CID spectra of compound 10 and 11 in cefdinir capsule.

Identification of other impurities in cefaclor

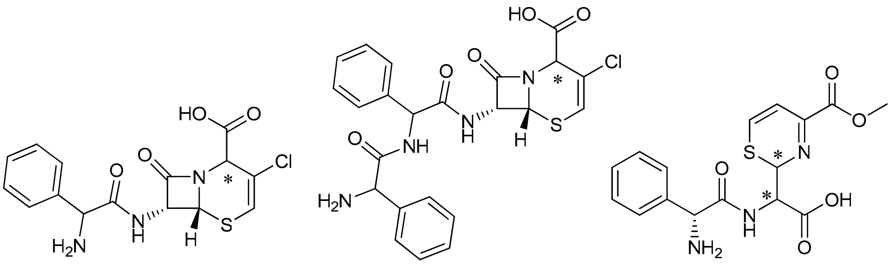

Scheme S-10: Structure of impurities identified in cefaclor capsule. A, compound 1 and 2; B, compound 14 and 15; C, compound 7 and 13

3.7.2.1 Identification of compound 11 and 12

Pseudo-molecular ion of both compound 11 and 12 were observed at *m/z* 400, which is 14 amu more than compound 10. The profiles of their CID spectra (Figure S-8) suggested they were ring-opened β-lactams. High yield of fragment formed from loss of hydrogen chloride (*m/z* 364) indicated a 5-chloride substituent and a delta-5 double bond on the thiazine ring. The high yield fragment formed from elimination of ammonium (*m/z* 347) indicated a primary amine located on the side chain, and their configurations of C-2 were the same. The neutral

loss of 32 (*m/z* 315 $[347-32]^+$, 100% for compound 11, 50% for compound 12) suggested that there was a methoxyl or a hydroxymethyl group. Compound 11 and compound 12 were identified to be methyl ester of compound 10. 2-((R)-2-amino-2-phenylacetamido)-2-(4R/S-5- chloro-4- (methoxycarbonyl)-3,4-dihydro-2H-1,3-thiazin-2-yl)acetic acid (Scheme S-11).

3.7.2.2 Identification of compound 14 and 15

Pseudo-molecular ions at *m/z* 501 suggested that the molecular weights of compound 14 and 15 were both 500 amu (Figure S-9), which is the same as N-phenylglycyl cefaclor, the impurity H of cefaclor . High yield of fragment at 368 formed in the ion source indicated both compounds were unstable, and there was an N-phenylglycyl substituent group localize on the molecule. Extensive fragmentation of the daughter ion *m/z* 368 leads to a spectrum which is the same as that of compound 1 and 2. Compound 14 and 15 were identified as N-phenylglycyl delta-3-cefaclor based on these observations (Scheme S-10 B).

3.7.2.3 Identification of compound 7 and 13

CID spectra of both compound 7 and 13 display protonated molecule at *m/z* 364, which is 36 amu less than compound 11 and compound 12 (Figure S-10). This observation suggested that compound 7 and 13 were hydrogen chloride elimination products of compound 14 or compound 15. It was confirmed by the low abundance of A+2 isotopic peak (3 -5 %) in the all scan spectra of them. The fragments originate from elimination of NH_3 (*m/z* 347 $[M+H-17]^+$ and *m/z* 303 $[320-17]^+$) indicated the existence of primary amine group on the side chain. The observations of elimination of methanol (*m/z* 288 $[320-32]^+$ and *m/z* 271 $[303-32]^+$) suggested the existence of methyl ester group on the molecule. Compound 7 and 13 were identified as 2-((R)-2-amino-2-phenylacetamido)-2-(2R/S-4-(methoxycarbonyl)-2H-1,3-thiazin-2-yl)acetic acid (Scheme S-10 C). The different yields of fragments formed from elimination of NH_3 or CO2 are proposed to be attributed to the stereo configuration of C-2 of the thiazine ring.

3.7.2.4 Determination of compound 3 and 6

The protonated compound 3 and compound 6 were observed at *m/z* 382 and *m/z* 396 respectively. Similar to compound 12, both CID spectra of compound 3 and compound 6 display prominent fragments at *m/z* 364. The neutral loss at 18 amu from compound 3 suggested that there was a hydroxyl group at C-5 of the thiazine ring, the

neutral fragment at 32 amu represents a methoxyl group on the thiazine ring of compound 6 accordingly. The elimination of channel for both compounds was the same as which is observed in the CID spectrum of compound 1 and 2. Based on these observations, compound 3 was identified to be 2-((R)-2-amino-2-phenylacetamido)-2-(5-hydroxyl-4-(carboxy)-3,4-dihydro-2H-1,3- thiazin-2-yl)acetic acid (Scheme S-11) and compound 6 was identified to be 2-((R)-2-amino-2-phenylacetamido)-2- (5-methoxyl-4-(methoxylcarbonyl)-3,4- dihydro-2H-1,3-thiazin-2-yl) acetic acid (Scheme S-11).

3.7.2.4 Determination of compound 4, 5 and 8

Compound 4 is an isomer of compound 3. The display of a prominent fragment formed from elimination of methanol in the CID spectrum suggesed that there was a methoxyl group and a delta-5 double bond localized on the thiazin ring. Compound 4 was suggested to be 2-((R)-2-amino-2-phenylacetamido)-2-(5-methoxy-4-(carboxy)-3,4-dihydro -2H-1,3-thiazin-2-yl)acetic acid based on these observations (Scheme S-11). Compound 5 is also isomer of compound 3 and 4. The exhibition of prominent fragmentation of decarboxylation suggested that there was a delta-4 double on the thiazin ring. Fragment at 308 formed from extensive fragmentation of daughter ion at *m/z* 338 suggests the methoxyl group on the ring. Compound 5 was suggest as 2-((R)-2-amino-2-phenylacetamido)-2-(5-methoxy-4-(carboxy)-3,6-dihydro-2H-1,3- thiazin-2-yl)acetic acid based on these data (Scheme S-11).

Compound 8 is an isomer of compound 6. Its CID spectrum displays prominent fragment at *m/z* 352 ($[M+H-44]^+$), while the fragment in the highest yield of compound 6 is at *m/z* 364 ($[M+H-32]^+$). This observation indicated that the double bond of compound 8 was between C-4 and C-5. Based on these data, compound 8 was proposed to be 2-((R)-2-amino-2-phenylacetamido)-2-(5-methoxyl-4-(methoxylcarbonyl) -3,6-dihydro-2H-1,3-thiazin-2-yl) acetic acid (Scheme S-11).

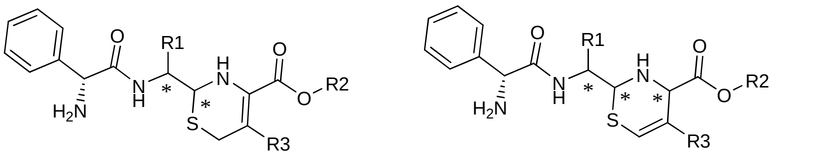

5, R1 = COOH, R2 = H, R3 = OCH_3
8, R1 = COOH, R2 = CH_3, R3 = OCH_3
9, R1 = COOH, R2 = H, R3 = Cl

3, R1 = COOH, R2 = CH_3, R3 = OH
4, R1 = COOH, R2 = H, R3 = OCH_3
6, R1 = COOH, R2 = CH_3, R3 = OCH_3
10, R1 = COOH, R2 = H, R3 = Cl
11, R1 = COOH, R2 = CH_3, R3 = Cl
12, R1 = COOH, R2 = CH_3, R3 = Cl

Scheme S-11: Impurities identified in cefaclor capsule.

these impurities, whose molecular weight is 18 amu more than that of cefaclor were possibly ring-opened cefaclor. There are two compound (compound 9 and 10) found in the HPLC MS data of cefaclor capsule. CID spectra of both compound 9 and 10 displays a similar profile as the ring-opened β-lactams (Figure S-2 F, Figure 9C and D) identified in cefixime and cefdinir. CID spectrum of compound 9 displays high yields of fragments formed from elimination of small molecules, e.g. H_2O, CO_2, NH_3 and HCl (*m/z* 306, 100%). These observations confirmed the structure of ring-opened cefaclor. The CID spectrum of compound 10 displays very high yield of fragment at *m/z* 306 (100%), but lower yield of fragments at *m/z* 342 $[M+H-44]^+$, *m/z* 325 $[342-17]^+$ and *m/z* 262 $[306-44]^+$. As described, localization of the double bond on the thiazine ring affects the yields of HCl (Scheme 9). The double bond of compound 9 was suggested to locate between C-4 and C-5 (delta-4), which of compound 10 locates between C-5 and C-6 (delta-5), based on these observations. Double bond at delta-5 is advantageous to eliminate the chloride at C-5, while delta-4 double bond is benefit decarboxylation at C-4 thereof. The low yield of fragment originates from elimination of CO_2 in the CID spectrum of compound 10 was suggested to be resulted from the delta-5 double bond on the thiazine ring. Based on these data, compound 10 was identified to be the delta-5-isomer of compound 9. Therefore, compound 9 was identified as 2-((R)-2-amino-2-phenylacetamido)-2-(5-chloro-4-(carboxy)-3,6- dihydro-2H-1,3-thiazin-2-yl)acetic acid, compound 10 was identified as 2-((R)-2-amino-2-phenylacetamido)-2-(4R/S-5-chloro-4-(carboxy)-3,4-dihydro-2H-1,3- thiazin-2-yl) acetic acid (Scheme S-11).

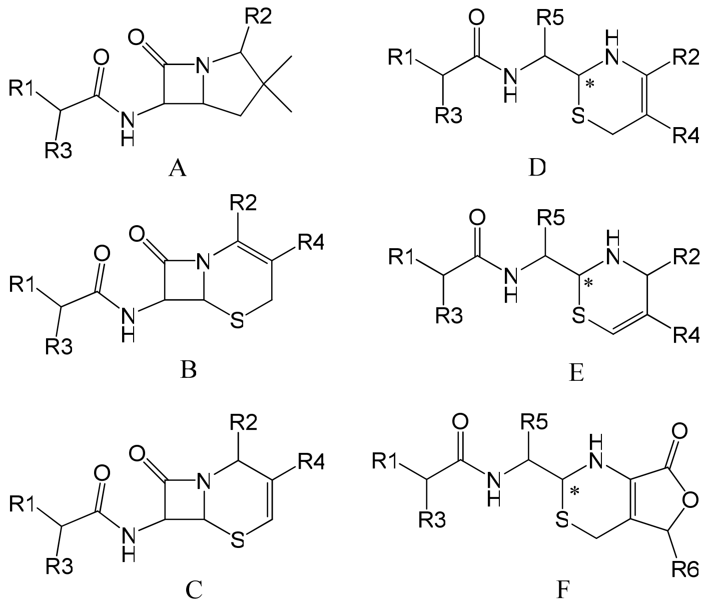

Scheme S-12: Representative structure of β-lactam antibiotics and their impurities.

Most β-lactam antibiotic consist a core of thiazole lactam (or thiazine lactam) and an amide side chain localized on the lactam ring (Scheme S-12). Generally, R1 is an aromatic group and R3 is a carboxyl group. Much diversity has been observed of R3 and R4. The diversity of the substituent groups on the thiazole (or thiazine ring) and the side chain are related to the antimicrobial spectra and stabilities. Impurities of a β-lactam antibiotic include process impurities, degradants, and possible isomers. R5 is a carboxyl produced from the hydrolysis of the β-lactam. However, it is eliminated in decarboxylated impurities. Degradants with furone ring usually formed when R4 is methene or a vinyl group. Diagnostic fragmentations of β-lactams and their impurities have been obtained under low energy activated CID experiment. These characteristics disappeared when a CID spectrum was obtained under high energy activation.

Generally, mass spectrometry is not good at differentiating isomers. However, several diagnostic fragmentations are observed to be useful to determine the localization of a substituent group, even the configuration of chiral carbon in this job. The localization of double bond on the thiazine ring, which is generally difficult to be determined using MS data, has been successfully determined based on the yields of elimination of hydrogen chloride of cefaclor impurities. For the impurities with β-lactam ring, the fragments originate from the cleavage of lactam ring is the best diagnostic characteristic to determine the localization of the substituents. Elimination of methanol, hydrogen chloride, as well as H_2O, was used to determine the localization of the double bond on the thiazine ring. A double bond at delta-3 is proved to be advantageous to the elimination of R4 when it is a group linked with a heteroatom, e.g. methoxyl, hydroxyl or chloride. The effect of the localization of the double bond on the thiazine ring was also observed for the ring-opened impurities. Cleavage of R3 is observed in some ring-opened impurities. Its yield is supposed to be related to the configuration of C-2 of the thiazine ring without the fused β-lactam. Unfortunately, we failed to find any reference compound to determine what configuration it represents.

It is unimaginable to interpret the MS data obtained with a single compound, because there should be several fragmentation channels which can explain the origination of a fragment. It is advisable to postulate the structure of a fragment based on the MS data from a series of homologues. HPLC MS analysis provided CID spectra of series structural related compounds. All of the fragmentation patterns proposed in this job, e.g. *m/z* 210 and *m/z* 337 of

cefixime, are based on observations in different compounds.

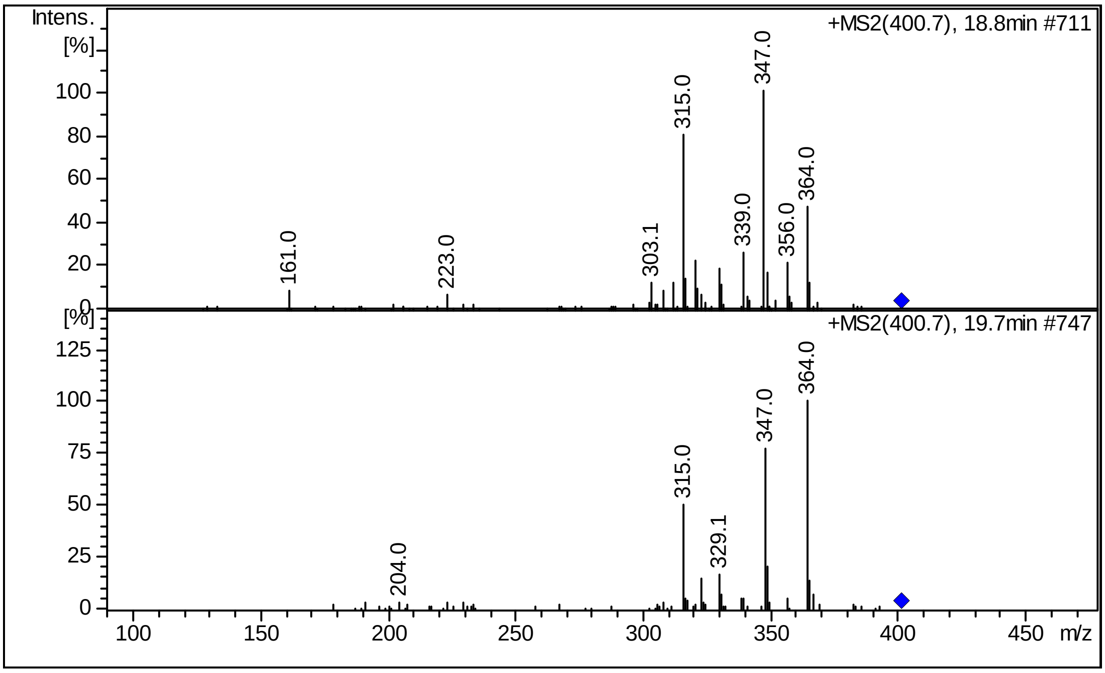

Figure S-8: CID spectra of compound 11 (upper) and compound 12 (lower) in cefaclor capsule.

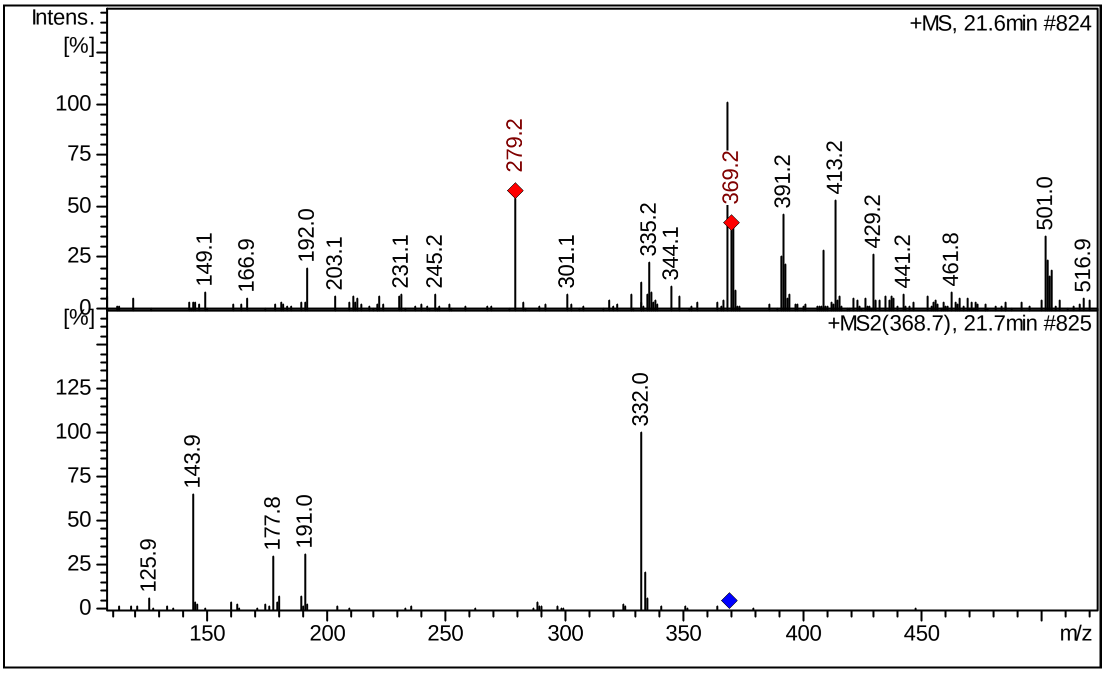

Figure S-9: CID spectra of compound 14 and 15. Upper, MS scan; Lower, CID spectrum of the in source formed daughter ion at m/z 368.

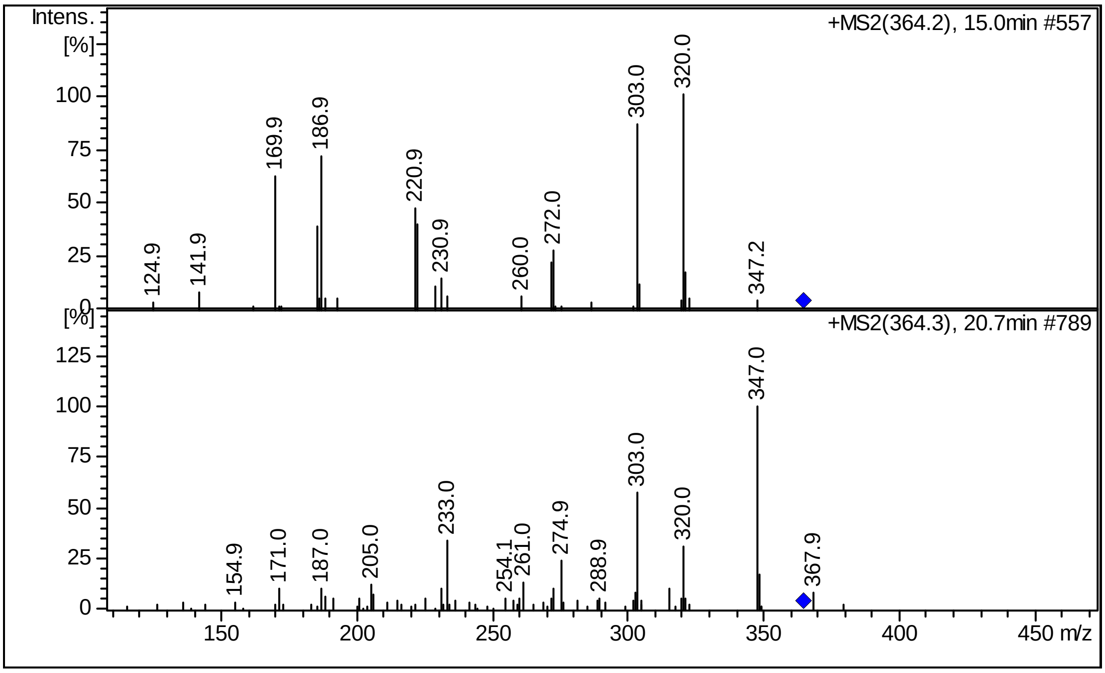

Figure S-10: CID spectra of compound 7 (upper) and compound 13 (lower) in cefaclor capsule.
